# Multi-Wave and Hybrid Imaging Techniques: A New Direction for Nondestructive Testing and Structural Health Monitoring

**DOI:** 10.3390/s131216146

**Published:** 2013-11-27

**Authors:** Yuhua Cheng, Yiming Deng, Jing Cao, Xin Xiong, Libing Bai, Zhaojun Li

**Affiliations:** 1 School of Automation Engineering, University of Electronic Science and Technology of China, Chengdu 610054, China; E-Mails: cjtobebest@126.com (J.C.); xiongxinuestc@163.com (X.X.); bailb991@163.com (L.B.); 2 Laboratory of Imaging and Intelligent Prognostics (LIIP), University of Colorado Denver, Denver, CO 80217, USA; 3 Electro-Motive Diesel, A Caterpillar Company, La Grange, IL 60525, USA; E-Mail: zjli@uw.edu

**Keywords:** structural health monitoring, nondestructive evaluation, multi-wave imaging, hybrid imaging

## Abstract

In this article, the state-of-the-art multi-wave and hybrid imaging techniques in the field of nondestructive evaluation and structural health monitoring were comprehensively reviewed. A new direction for assessment and health monitoring of various structures by capitalizing the advantages of those imaging methods was discussed. Although sharing similar system configurations, the imaging physics and principles of multi-wave phenomena and hybrid imaging methods are inherently different. After a brief introduction of nondestructive evaluation (NDE), structure health monitoring (SHM) and their related challenges, several recent advances that have significantly extended imaging methods from laboratory development into practical applications were summarized, followed by conclusions and discussion on future directions.

## 1. Introduction

With the rapid development and inevitable aging of infrastructures, it is critical to monitor structural health and ensure the system's integrity by detecting the onset of damages, for example, fatigue cracks and corrosion in critical structures, such as ship hulls, off-shore oil and gas production facilities, power plants, marine structures, rails and multi-layer airframe structures. The development of imaging techniques for evaluating these damages provides direct or indirect, real-time and quantitative results, many of which are well-known to the experts in the nondestructive evaluation (NDE) fields that include, but are not limited to, X-rays and gamma-rays, microwave, eddy current, acoustic, optical and thermal imaging methods. Each individual technique has its own and specific applications, advantages, as well as limitations, due to the nature of imaging physics. For example, conventional electromagnetic imaging can work in three different regimes of resolution: coherent or far-field, diffusive and near-field. There is always a trade off between the imaging depth and spatial resolution, due to the physical limits and actual set up of the system. It is only in the far-field that wavelength determines resolution, which is, however, at the order of the transducer size and independent of wavelength in the near-field regime. Recent development in other single imaging methods is trying to achieve improved imaging quality and better detectability, e.g., sonic imaging is used for detecting defects or hidden objects inside the structure [1], thermal imaging methods proved capable of mapping the length and shape of cracks and provides qualitative information about crack depth [2]. In order to find a way to improve the imaging capability of each single imaging technique and to reach beyond the physical diffraction limit, the multi-wave imaging concept was proposed in recent years [3–6], which consists of combining two different waves that were generated sequentially and taking advantage of the merit of each wave. Take thermal-acoustic imaging, as an example: acoustic waves provide image contrast and thermal absorption provides superior spatial resolution. Because of the way the waves are combined, multi-wave imaging may produce a single, but enhanced, image with the best contrast and resolution. Hybrid imaging shares the similar goal of enhancing the image quality, but by simply combining two single methods, and further, data fusion and image registration are typically necessary. In hybrid imaging methods, one wave does not serve as the generating source for the other wave type. One classical example is Positron Emission Tomography and Computed Tomography (PET/CT) imaging for clinical diagnosis. Multi-wave imaging, along with hybrid imaging, is now a fertile field from which new ideas and technologies are emerging. It is a new direction and has a bright future in NDEand structure health monitoring (SHM). The motivation of this review article is to summarize the development of both multi-wave and hybrid imaging methods in the nondestructive testing and structural health monitoring community, since we have realized several research groups are pioneering to combine different forms of energies, such as electromagnetic/thermal, acoustic/thermal waves and optical/acoustic, to explore super-resolution and super-contrast imaging techniques; however, a systematic study and comprehensive review is lacking for this new direction in NDE and SHM imaging.

## 2. Relationships between Multi-Wave and Hybrid Imaging

SHM and its related NDE approaches are becoming increasingly important for maintaining the safety and integrity of infrastructures and complex systems that are configured with sophisticated data systems for electronics, propulsion, controls and other critical subsystems. In recent years, an increasing emphasis has been placed on the potential for using these data capabilities, in conjunction with emerging sensor and data management technologies for *in situ* health monitoring of material condition. However, SHM/NDE for the identification and characterization of structural degradation presents unique challenges. An understanding of potential damage mechanisms, structural design criteria, fail-safe features and structural maintenance philosophy is needed to develop a sensor-based system, so that the structural condition can be effectively monitored. Another challenge is how to provide real-time, accurate and quantitative image information about the damage.

The concept of multi-wave imaging was proposed independently by various groups in the physics community, in which one form of wave energy serves as the excitation source of the other. For example, in thermo-inductive imaging, absorbed electromagnetic radiation by induction causes a transient change in temperature that can be inspected by transient infrared thermography. Because of the way the waves are combined, multi-wave imaging has the potential to produce a single image with better contrast and higher spatial or spectral resolutions. Although it was initially introduced as a way to improve biomedical diagnostic capabilities [3], multi-wave imaging opens new avenues in the fields of SHM and NDE and provides a new direction for quantitative imaging, due to its superior outcomes compared to conventional imaging results.

As mentioned before, the multi-wave phenomenon is based on the effect that the interaction of one kind of wave with objects under testing can generate a second kind of wave, while hybrid imaging has a broader definition that strategically combines multiple imaging methods to achieve better fusion of information or, specifically, better detection in NDE and SHM problems. One success in the medical imaging society is the wide use of PET/CT imaging and the forthcoming PET/MR hybrid systems that give both abundant functional and anatomical details of the object. Generally speaking, the combination of different imaging techniques into one hybrid platform is beneficial to the imaging quality and speed and even makes simultaneous data acquisition possible. With minimal effort on image registration, the following data analysis and post-processing can be significantly eased compared to individual image acquisition. For NDE imaging, one example is magneto-optic imaging (MOI), which is based on the Faraday rotation effect and the interaction between electromagnetic waves and optical waves. This integration of both EM and optics generates real-time images with high sensitivity, since the optical rotation angle is proportional to the local small electromagnetic field. There is no doubt that multi-wave imaging and hybrid imaging are emerging as solutions to those SHM/NDE challenges.

## 3. Multi-Wave Phenomena and Imaging

For SHM and NDE applications, various techniques based on the multi-wave phenomena have been proposed, studied and developed, being successful as detection methods, although with limited success in quantitative imaging, such as electromagnetic-thermal methods, electromagnetic-ultrasonic methods, ultrasonic infrared thermal wave methods, photo-thermal methods and photo-acoustic methods, to name a few. This section provides a comprehensive review on the multi-wave methods for SHM/NDE and discusses their advantages and limitations, as well as the hurdles and the potential for future development.

### 3.1. Electromagnetic-Thermal Methods

Electromagnetic-thermal (EM-T) nondestructive inspection has been proposed as an alternative to the classical eddy current (EC) testing for just more than a decade [7]. This technique, also known as eddy current thermography [8–11], tone burst eddy current thermography (TBET) [12–14], thermo-inductive inspection and induction thermography [15–17], combines electromagnetic illumination of the work-piece, heating up of the material by induction and imaging by transient infrared thermography to provide a fast and efficient method for defect detection and material characterization over a relatively large area. Thermographic images picked up by an infrared (IR) camera can be evaluated to figure out the major defects, and the data can be further processed to provide quantitative information about defects. Pulsed eddy current (PEC)-stimulated thermography by combining PEC and thermal cameras has also been investigated recently as one of the electromagnetic-thermal methods [18–20]. The method injects a short pulse of current (typically less than 1 s) with high intensity into the samples under test and then obtains images from an infrared camera. Since the broadband eddy current can penetrate deep into the conductive materials, EM-T techniques can detect both surface and subsurface anomalies, even the hidden defects in complex components.

In 2006, Oswald-Tranta and Wally modeled the eddy current distribution inside the material and investigated the temperature distribution around a crack with different penetration depths using finite element modeling (FEM) and experiments with metallic materials [15]. For a surface crack with a depth of 1 mm, the calculated temperature distributions around the surface crack are depicted in Figure la,b for different penetration depths of 1 mm and 0.1 mm, respectively. Results show that the tip of the crack is warmer, whilst the edge of the crack is colder than the surface of the material after a very short heating duration; see Figure la. Conversely, if the penetration depth of the eddy current is much smaller than the depth of the crack, the edge of the crack is warmer after a very short heating duration; see Figure lb.

Another study regarding thermographic crack detection by eddy current excitation was carried out by Zenzinger *et al.*, and it described a phase algorithm to increase the sensitivity of small defects [8]. This paper concluded with an indication that the simulation calculations and resulting coil designs would decisively determine the future application spectrum of eddy current thermography.

**Figure 2.**
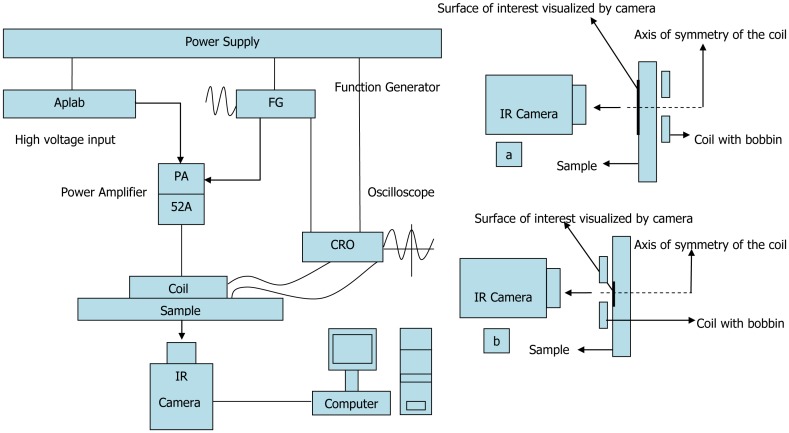
The experimental apparatus of tone burst eddy current thermography (TBET) in schematic format on the left and the two modes of data collection, *i.e.*, transmission and reflection, on the right-hand side.

**Figure 3.**
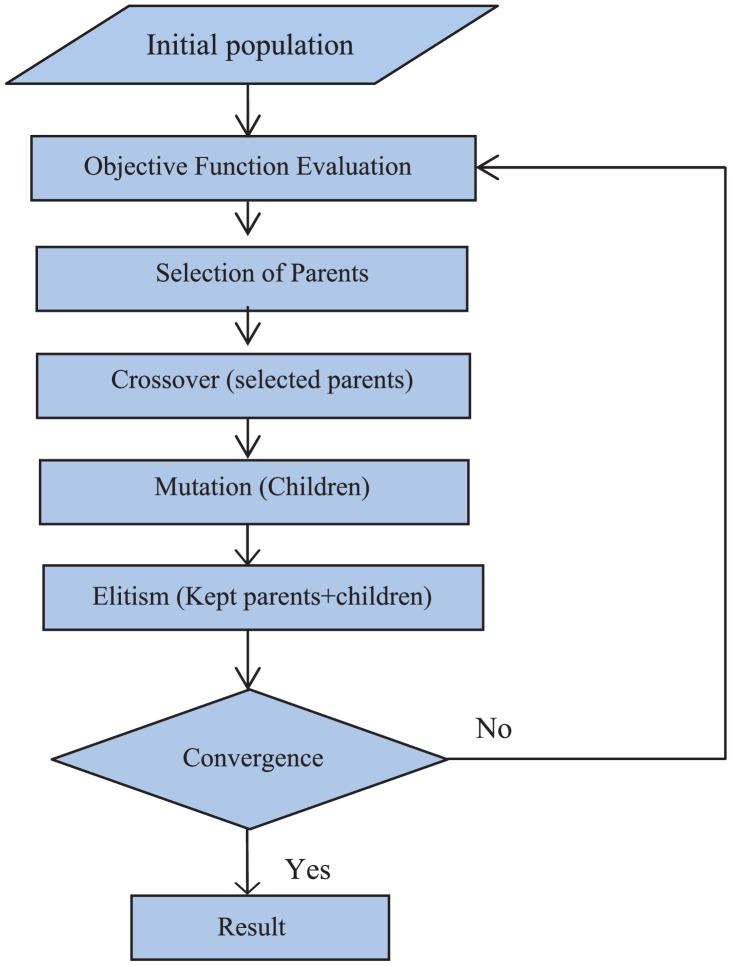
Flow chart showing the genetics algorithm (GA)-based inversion method.

Tone burst eddy current thermography (TBET) [12,13], which employs surface heating with the use of tone burst (a fixed number of cycles) ACpulses, was explored in 2008. In the paper published by Kumar *et al.*, they discussed the applications of TBET and compared it with conventional thermography techniques [12]. The typical apparatus of TBET is illustrated in Figure 2. Krishnamurthy *et al.* [13] further investigated the optimum frequency (peak frequency) of eddy current excitation, which would give a maximum temperature increase for a given thickness. The simulation was done by COMSOLmulti physics software to study the peak frequency values for different thickness, electrical conductivity and the thermal response of the sample (both plate and pipe geometries). The validity of the finite element (FE) model was verified by the good correlation between simulation and experimental results. Besides, a proof-of-concept demonstration of inverse analysis for determination of defect size (radius and depth) in metals was published in 2012 [14]. The inversion of the TBET data was executed with the use of the genetic algorithm (GA)-based inversion method, which can be summarized as shown in Figure 3.

Simulations were performed using FEM to obtain the temperature data, which were then used to reconstruct the radius and depth of the wall thinning defects in an aluminum plate with the inversion method. The analysis involved two cases of defect radii, one larger than the coil inner radius and the other smaller than the coil inner radius. It showed that the smaller size of the coil would improve the sizing of the defects, but a tradeoff must be considered for the decreased scan efficiency.

Considering that the shape of the coil in EM-T NDTaffects the eddy-current distribution on the plate, thus the distribution of the Joule heat, it is evident that temperature gradients also vary with the types of coils. Apart from the widely-used circular coils [21], N. Tsopelas and N.J. Siakavellas employed square coils, planar circular and planar square ones [22]. It has been shown that the coils considered have similar efficiency in crack detection at the optimum distance, which is equal to 1/4 of the diameter for a circular coil or 1/4 of the side for a square coil.

**Figure 4.**
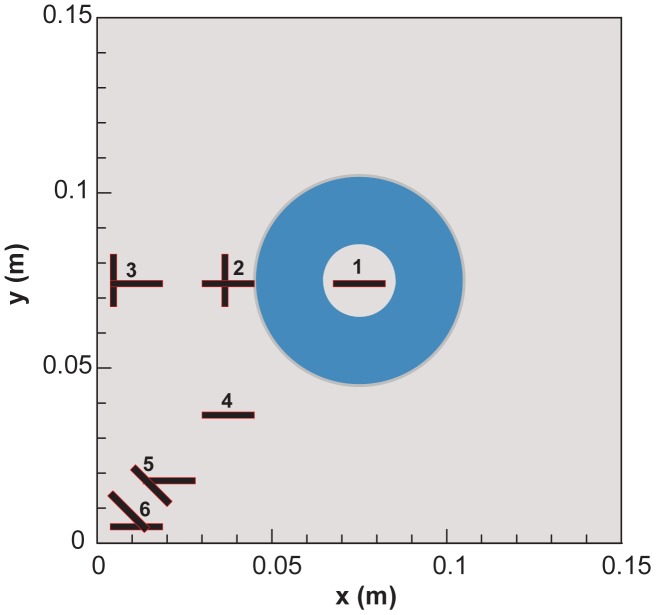
Crack positions in the plate (the coil is placed above the plate center at the optimum distance, *i.e.*, z =10 mm).

However, when the coils and plate were in close proximity, results differed, and the planar coils performed better. In 2009, they further investigated whether an appropriate analysis of the numerical results could make improvements in identifying the position and the shape of ambiguous cracks [23], with the purpose of determining the probability of reducing the total number of inspections. They employed three techniques—image subtraction, depiction of the norm of the spatial derivatives of temperature and the discrete Fourier transform (DFT). Figure 4 illustrates the six different positions of a crack considered in the study. The investigation showed that data processing did improve the detection of cracks significantly, but the performances varied according to the technique used.

On the basis of previous studies, they also investigated and verified the performance of the EM-T method in detecting cracks with circular aluminum plates [9]. The published paper focused mainly on the crack orientation with respect to the current flows and heat flows, which affect the effectiveness of the method, and researchers tried to find out when they should implement longer periods and/or regions. The experiments concerned the detection of the crack in six circular aluminum plates at various positions and orientations. It showed that Fourier transform improved the detection of cracks perpendicular to the current flow considerably. Additionally, improvements in numerical results for the cracks perpendicular to the heat flow could be achieved by both the norms of the spatial derivative of temperature and Fourier transform. Furthermore, they experimented with square aluminum plates [10] in 2011 to study the performance of this system. The group intended to investigate the effectiveness of the method with higher excitation frequencies and 3D work-pieces in the near future.

**Figure 5.**
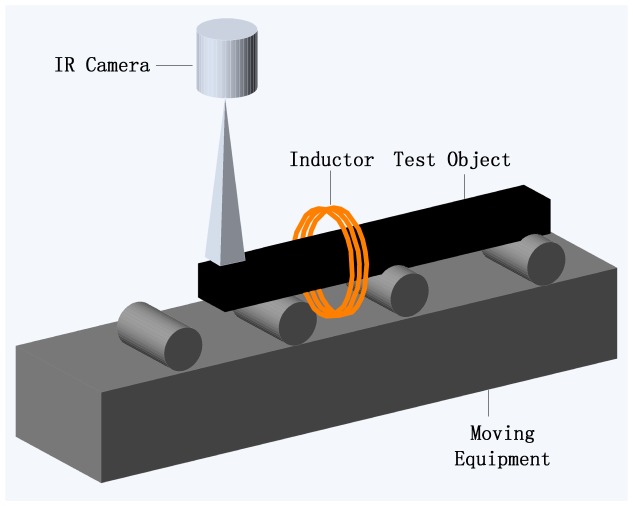
Experimental setup with the inductor controlled by moving equipment.

**Figure 6.**
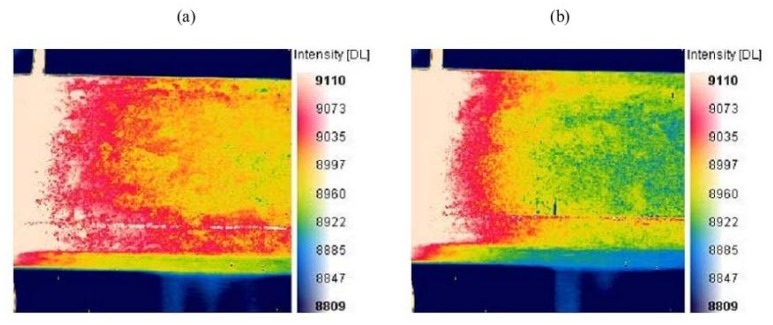
Experimental results of a real test object with the cross-sectional area of 50 × 50 mm (**a**) without water and (**b**) with water (Reproduced from [16] with permission).

Noethen *et al.* [16] carried out thermo-inductive measurements on ferritic and austenitic steels in 2010. The metal test parts move through the inductor controlled by moving equipment; see Figure 5. The uniqueness of their work is that a very thin distilled water film is used to superimpose the surface of different samples, including artificial and real test ones. It was presented that the water homogenized the emissivity of the oxidized surface, as illustrated in Figure 6, and that the amount of water affected the resulting image. The efforts carried out by Chen *et al.* [24] focused not only on the surface and sub-surface flaws, but also on the rebar detection.

**Figure 7.**
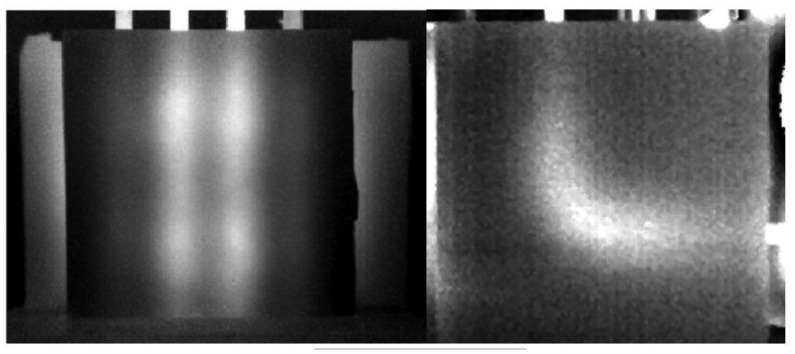
Thermal image for concrete samples using electro-thermography technique to detect rebar (Reproduced from [24] with permission).

Figure 7 shows the result of the thermography inspection for two concrete samples with different shapes of rebar.

Although there has been a considerable amount of literature on the research of eddy-current thermography, these studies appear not to pay much attention to the probability of detection(POD) for eddy-therm inspection. A paper relevant to this research was published by Weekes and his colleagues [25], which managed to establish the probability of detecting cracks as a function of crack length in laboratory-type metal beam specimens. Fatigue cracks in steel, titanium and waspaloy were firstly quantified by a signal process, and the acquired data were then moved into a cumulative log-normal probability of the detection model. The eddy-therm POD results for the detection of fatigue cracks in each of the samples were compared to results from POD studies of alternative NDE methods, proving its good detectability in metals.

The EM-T technique has also found application in health investigations on different components of automotive and aeronautic industries. Besides those efforts on metallic materials, research on the crack detection of carbon fiber reinforced polymer (CFRP) materials have also progressed in recent years. A 3D finite-element model based on shell elements, which was derived from Whitney's elements [26], was developed by Ramdane *et al.* [17] to help optimize the detection and characterization of defects. The anisotropy, nonlinearity of materials and the presence of thin regions were taken into account in the model. Numerical simulations and experiments on metallic and composite materials showed that cracks in CFRP could be detected using a thermo-inductive technique.

**Figure 8.**
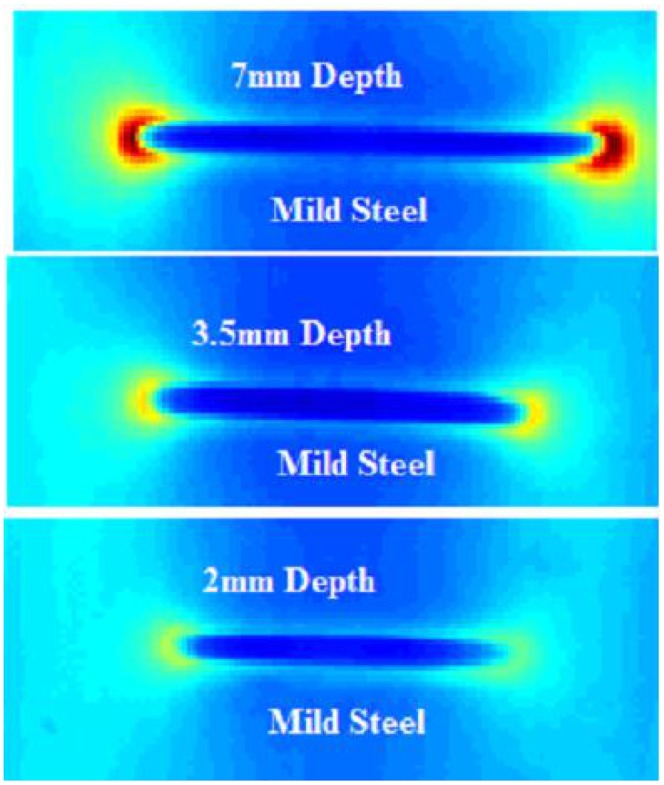
Pulsed eddy current (PEC) thermography image of a steel sample with different defects (Reproduced from [19] with permission). The sample is 40 mm × 30 mm × 10 mm, and the defects are, respectively, 15 mm × 2 mm × 7 mm, 10 mm × 2 mm × 3.5 mm, 10 mm × 2 mm × 2 mm.

For PEC thermography, the group led by G.Y. Tian is one of the pioneering groups in the UK in conducting inspection and imaging research. In 2011, they carried out research studies to investigate the detection of corrosion in structural steel components by PEC thermography [18]. Changes caused by corrosion in electrical conductivity, permeability, thermal conductivity, heat capacity, depth and density and their influences were considered in the analysis. Their work included analysis of surface thermal images, as well as experimental studies with structural steel (S275) samples, and the results indicated the effectiveness of PEC thermography for corrosion detection and characterization. In the same year, PEC thermography was implemented for the first time to detect notches (surface cracks over the full width of the sample, but finite in depth and width) in CFRP samples [27]. The position invariance of the coil with respect to the notch along the fiber direction was also studied in the experiment. Meanwhile, another paper dedicated to the understanding of depth and the tip effect on PEC thermography was published in 2011 [19]. The experimental results of three defects with different depths on the mild steel sample can be seen in Figure 8. The group believed that the research of transient heating propagation and magnetic flux distribution would hopefully provide useful information for feature extraction and pattern recognition techniques in the quantitative analysis of PEC thermography.

**Figure 9.**
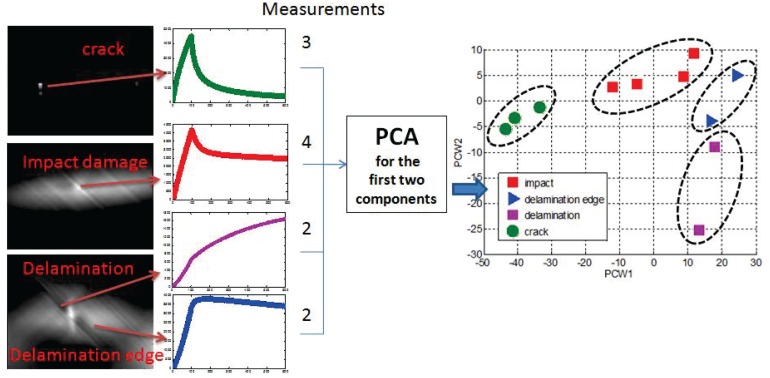
Principal component analysis (PCA) classification for cracks, impact damages and delaminations (Reproduced from [20] with permission).

Cheng and Tian built a PEC-stimulated thermography system at NUAA [28], which was extended from previous joint work between Newcastle and Bath Universities. In the paper, PEC-stimulated thermography was compared with other NDT methods, such as ultrasonic (UT), flash thermography and eddy current (EC) scanning, in the detection of man-made, dedicated delaminations with varied diameters and depths. From the results obtained, it seemed that PEC-stimulated thermography had a worse detection ability than UT scanning in detected depth, but performed particularly well in fiber orientation evaluation. In general, PEC-stimulated thermography provided a good alternative for CFRP delamination detection. Moreover, the group used this technique to characterize three types of common defects in CFRP composites—cracks, impact damage and delamination [20]. The different heating patterns and transient temperature responses can help to fix defects and study the physical property variation with the principal component analysis (PCA), as shown in Figure 9. Results indicated the relationships between electrical and thermal conductivity distribution and impact energy: electrical conductivity in the impact area decreases and thermal conductivity increases with the increase of impact energy (not large enough to generate a surface crack).

In 2012, Abidin *et al.* [11] discussed the advantages provided by the EM-T technique and its applications along with results from 3D FEM numerical simulations and experimental investigation on in-service samples. Numerical results demonstrated that the angular characteristic of a defect will influence the overall magnetic field distribution and the temperature distribution resulting from interaction between eddy currents and defects. Experimental investigation on a rail head sample with real defects also proved the effectiveness of eddy current thermography in providing comprehensive and reliable defect assessment. In conclusion, the EM-T method combines the advantages of eddy current inspection for the detection of buried cracks with the advantages of thermography to make it a fast, contact-free imaging technique. The method utilizes the high performance of eddy current testing without the known problem of the edge effect, especially for components of complex geometry. It provides a reliable and efficient method for defect detection and characterization over a relatively large area. It is efficient for the effective detection and measurement of multiple, natural defects inside in-service components, even hidden subsurface defects.

### 3.2. Electromagnetic-Acoustic Methods

**Figure 10.**
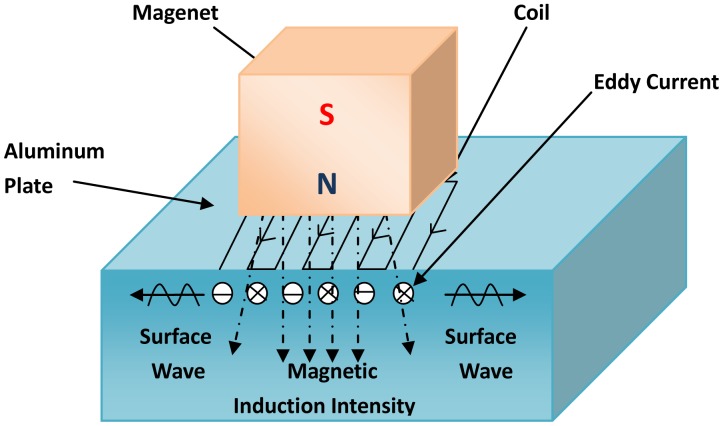
Schematic diagram of an electromagnetic acoustic transducer (EMATS).

Electromagnetic-acoustic (EM-A) techniques emerged in the middle-late part of the last century as a new ultrasonic testing method and were then applied to nondestructive testing [29]. This mainly refers to the electromagnetic acoustic transducers (EMATS) technique, and EMATs play a major role in the testing system. As the non-contact ultrasonic transmitting and receiving device [30] used for the nondestructive inspection and materials characterization of conductive materials, EMATs generate and detect ultrasonic waves via electromagnetic coupling between the transducer and the samples. The schematic diagram of an EMAT is shown in Figure 10. Usually, the EM-A system consists of a magnet, a coil and a specimen, in which the coil and magnet are usually regarded as an EMAT probe. This technique has many advantages, such as being free of a couplant, having no need of surface preconditioning of the test piece, non-contact operation and high temperature operation. What is more, various ultrasound waves can be used in this technique, like surface waves, plane waves, bulk waves, *etc.* [31,32].

**Figure 11.**
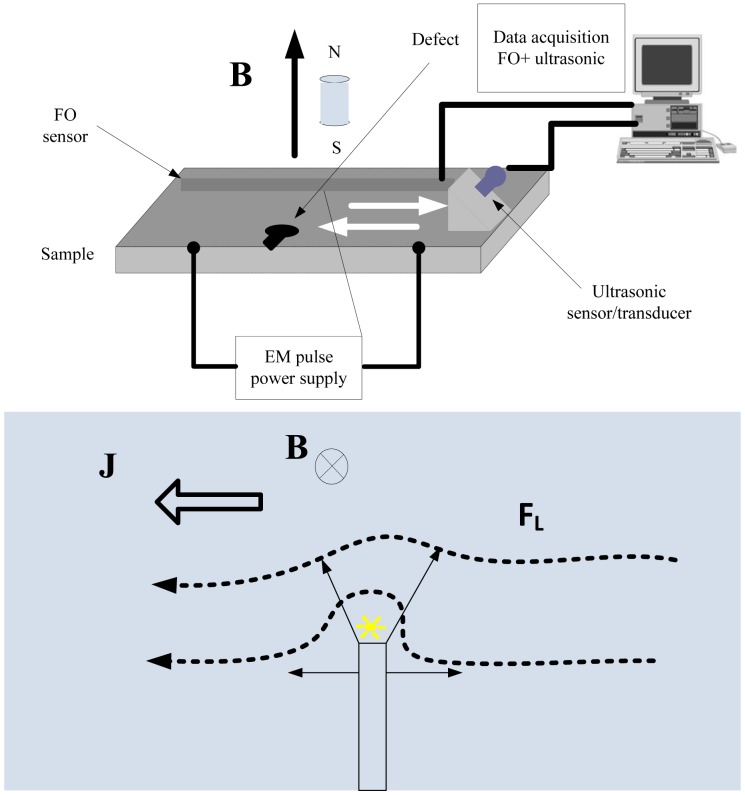
Experimental setup and schematic of the EMAT method.

Finkel and Godinez demonstrated the ability and advantages of the EM-induced defect stimulation to identify small cracks and ferromagnetic inclusions in thin-walled aluminum structures [33]. A method based on electromagnetic modulation of the ultrasonic signal was also proposed in the paper. The method could increase the detection ability of small fatigue cracks compared with the crack closure technique proven by Nagy [34]. The experimental setup and schematic of the method is illustrated in Figure 11. It shows that an EM pulse was able to induce and stimulate elastic waves by the defect itself and can used for modulation of an ultrasonic signal.

MacLauchlan *et al.* summarized the advantages and recent significant advancements in three-dimensional electromagnetic and ultrasonic modeling, magnet materials, analog electronics, phased array instruments and digital signal processing, which had lead to a substantial improvement in the performance of EMATs [35]. A 32 active channel phased array system was used to investigate the inspection of submerged arc welds (SAW) during welding and showed good performance.

It is widely known that EMATs operate on ferromagnetic materials via two different transduction mechanisms: the Lorentz force and magnetostriction. The Lorentz force is in the position of the dominant transduction effect and is not significantly sensitive to the typical range of the physical properties of steel. Experimental tests and numerical simulations undertaken by Ribichini *et al.* in 2012 [36] indicated that the Lorentz force was the largest transduction mechanism on steel materials, regardless of the magnitude and direction of magnetic bias the field employed, while the Lorentz force and magnetostriction were of the same order in nickel. The magnetostrictive sensor technology based on magnetostriction was developed by Southwest Research Institute (SwRI) for long-range inspection and structural health monitoring of pipes, plates, bridge cables and tubes. It was fast and cost-effective.

**Figure 12.**
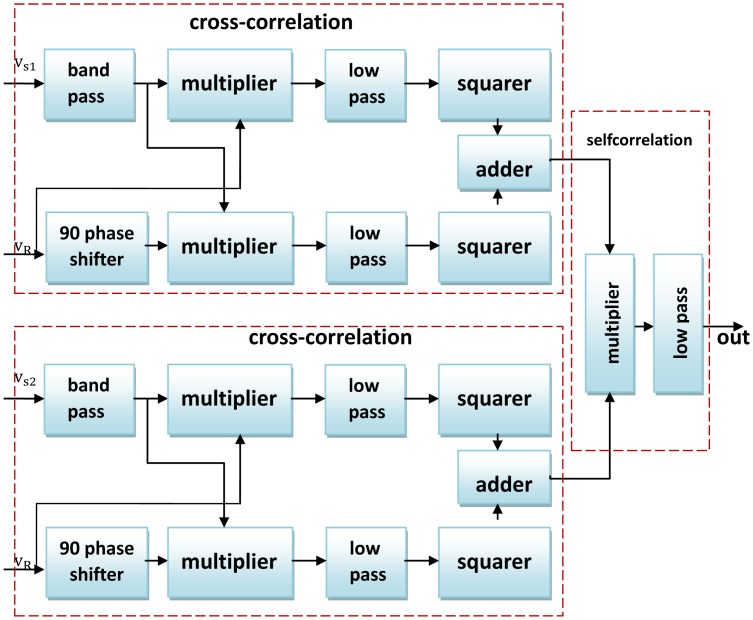
Schematic diagram of cross-and-self-correlation detection.

Considering the poor transduction efficiency of EMATs and its high sensitivity to surrounding electrical noise, an electromagnetic ultrasonic inspection system must possess the strong ability of weak signal detection, so as to extract defect information from received signals. In 2008, Lei *et al.* proposed a weak signal detection technique based on moving average, cross-correlation and self-correlation methods to extract effective information of the acquired signals [37]. The schematic diagram of the proposed technique is shown in Figure 12, combining both the cross-correlation and self-correlation method. The feasibility and efficiency of the technique was verified by experimental results with high reproducibility.

**Figure 13.**
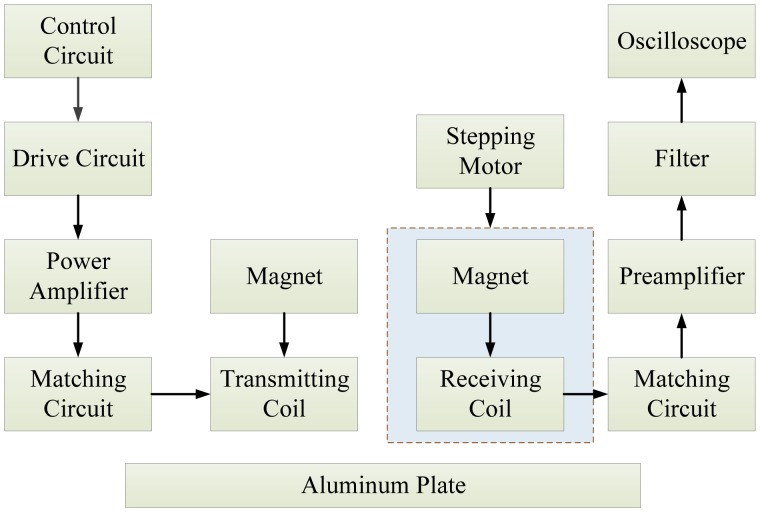
Schematic of the experiment for verifying the relationship between lift-off distance (changed by stepping motor) and transduction efficiency

Although EMATs have several distinct advantages, the transduction efficiency of the transducers is very poor, because of the shortcoming of design theories of EMATs. Considering that the relationship between the parameters of an electromagnetic surface acoustic transducer and its transduction efficiency has been seldom reported, Wang *et al.* [32] studied the transduction efficiency characteristic of the transducer based on the analysis of their EMAT model to establish a theoretical foundation in 2009. Figure 13 shows the diagram of the experiment aimed at verifying the relationship between lift-off distance and transduction efficiency The experimental results indicated that the decrease of lift-off distance, coil conductor width, magnet length and width and the increase of magnet thickness could effectively improve the transduction efficiency of the EMAT.

In 2010, another paper dealt with the design of electrical parameters, and a geometric parameters of electromagnetic acoustic surface wave detection system was published by Yang *et al.* [29]. The directory information provided by this design could serve as a key basis for an automatic flaw detection system for moving-wheels.

**Figure 14.**
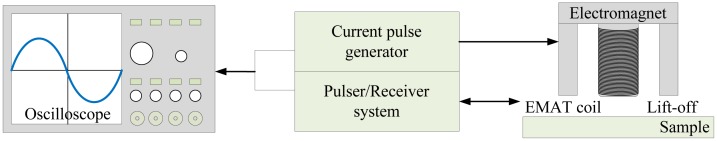
Schematic diagram of experimental electromagnet EMAT set-up.

Previous application of EMATs took advantage of small and compact permanent magnets to provide the high magnetic field required for detection, but the low Curie point of the permanent magnet made a water cooling device necessary at the EMAT head and impeded the application of the permanent magnet. In 2009, Palmer *et al.* employed a pulsed electromagnet to provide the magnetic field [38]. The experimental set-up is illustrated in Figure 14. The results demonstrated a significant enhancement in the generated ultrasonic signal amplitude for operation on mild steel samples.

The paper published by Zhai *et al.* focused on the exciting equation, which had been set up from the relationship between the primary design parameters of EMAT and the characteristic equation of the Lamb wave. According to the equation, the excitation of the electromagnetic ultrasonic Lamb wave has something to do with three parameters: the meander-line coil spacing interval between adjacent wires, the frequency of the pulse current and the thickness of the specimen. They found the key to the optimization and proposed a method for optimizing excitation by removing the effect of multi-modes and dispersion [39].

**Figure 15.**
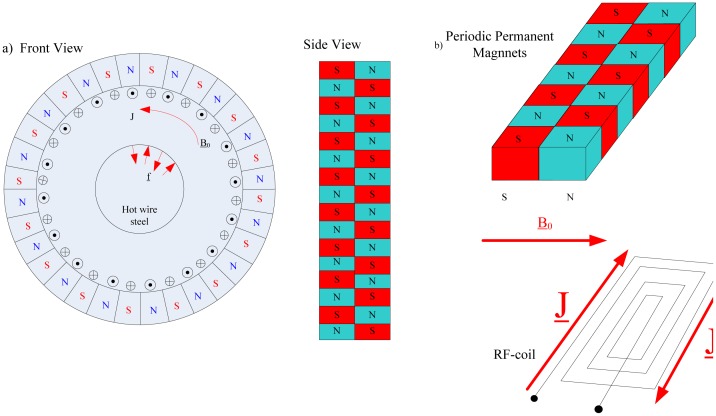
**(a)** Sketch of a Rayleigh wave EMUS probe for hot wire inspection; (**b**) sketch of a periodic permanent magnet to generate the magnetostatic field and an RF-coil to excite a transient eddy current.

Based on the INCOSTEELproject sponsored by the European Commission, a paper relevant to the creative and advanced EM-A techniques for the in-line inspection of hot wire steel was published by Marklein and Rahman in 2006 [40]. Two types of sensor techniques—the eddy current (EC) sensor technique and the electromagnetic ultrasonic (EMUS) sensor technique—were employed, respectively, to detect the surface defects and longitudinal cracks in this inspection procedure. A combination of both sensors was a good choice for detecting defects in hostile environments. Figure 15 displays a schematic view of an EMUS sensor operating with Rayleigh surface waves. To solve the modeling problem, various numerical methods, such as the finite integration technique (FIT) for the EMUS transducer, the finite element method (FEM) and the boundary element method (BEM) for the eddy current were chosen.

**Figure 16.**
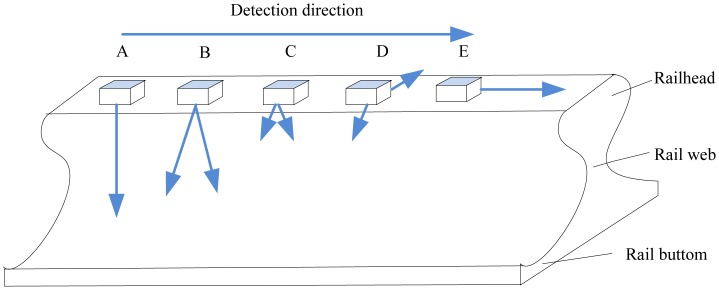
Placement of EMATs on the railhead.

The EMATs can also be used in railway structural health monitoring. The Scientific-Production Enterprise *VIGOR* proposed a rail flaw detection system UDCECRWTC01 [41], in which EMATs were used to generate a surface wave at the frequency of 0.25 MHz and 0.5 MHz, a 40° oblique incidence shear wave at 1 MHz and a 0° normal incidence shear wave at 1.8 MHz. Later, in 2010, Zhu *et al.* developed a rail flaw detection system based on multi-channel electromagnetic acoustic transducers under the control of DSP [42]. Because of the fact that a single transducer could not detect various flaws in a complicated rail structure, five channel transducers were used to detect all rail flaws—a 0° transducer, a 37° transducer, two 60° transducers and a surface wave transducer—which is depicted in Figure 16. Both the cumulative average method and cross-correlation detection method were used to realize the processing of the echo signals. The detection system achieved a standout denoising effect with high speed and realized the overall detection of the rails.

#### 3.2.1. Ultrasonic Infrared Thermal Wave Method

**Figure 17.**
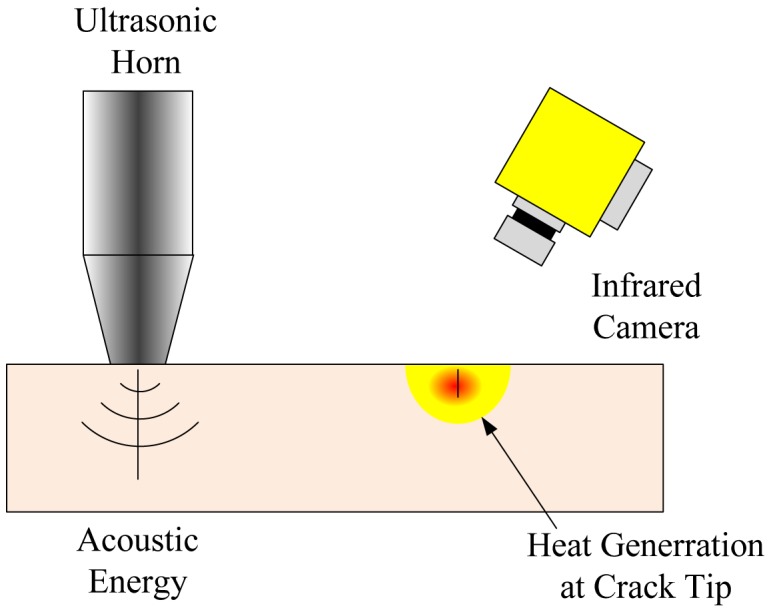
Principle of crack detection by the sonic infrared (IR) technique.

Ultrasonic infrared thermal wave nondestructive testing technology, which combines ultrasonic vibration excitation and infrared imaging technology, is used for the detection of defects, such as cracks and disband/delaminations on a variety of different materials and structures. This method is unique compared to other thermal methods, since the excitation source is not a heat source, but a sonic one. The testing technology is variously described in the literature as sonic infrared (Sonic-IR/SIR) [43–45], thermo-sonic [46,47], acoustic thermography [48], *etc.* The principle of crack detection by the sonic-IR technique [49] is shown in Figure 17, in which a short burst of high power acoustical energy is launched by an ultrasonic emitter. If there is a defect, such as a crack, inside the sample, the acoustical energy will induce vibrations and cause the crack interfaces to rub, then frictional heating is generated with the localized temperature increase. An infrared camera images the returning thermal wave reflections from the sample for further study to characterize the cracks. This technique has significant advantages and improvements over traditional NDE techniques, such as ultrasonic testing, liquid penetrant testing and eddy current testing. It is an effective, fast, wide-area and truly dark-field NDE/SHM method, since only the defects respond to the excitation. Both surface and subsurface cracks can be detected, especially small cracks, like stress corrosion cracking.

The sonic IR technique is a relatively new and thermal-based method for NDE developed by researchers at Wayne State University [50]. The method used a single short pulse of low frequency sound to serve as the excitation. The image of the crack appeared at the millisecond (ms) level, namely, the crack was visible in real time in the raw IR images, so that image processing or averaging was not necessary [47]. A variety of defects in several different materials were detected to display the capability of the sonic/ultrasonic IR technique to image subsurface impact damage, kissing disband and adhesion defects. Other examples of cracks in aluminum and titanium fatigue specimens were carried in the same year [46]. Experimental results showed that the sonic IR technique could inspect fatigue cracks as short as 20 micrometers in metal samples.

**Figure 18.**
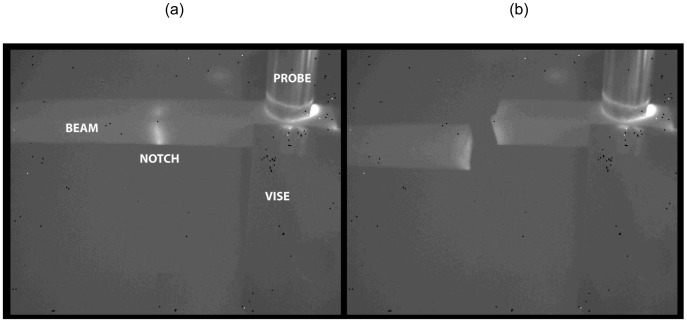
Photograph of (**a**) the thermal signature from the cracked beam couponand (**b**) the eventual fracture of the cracked beam coupon (reproduced from [43] with permission).

Meanwhile, the Lawrence Livermore National Laboratory (LLNL) carried out experiments to observe the capability of sonic IR to detect small cracks on several materials and flaw types [43]. Experimental results showed that the method and equipment used here were effective only in certain circumstances, but not in others. Excellent noticeable thermal images were produced only in the notched beam coupon specimens, while surface ground and Vickers coupons showed the inability to detect the flaws despite that man-made damage was plainly evident. Several parts smashed during testing, probably by being forced at resonance by the 20 kHz acoustic probe, as illustrated in Figure 18. Moreover, the response was discovered to be modestly dependent on the contact location of the acoustic probe, as well as on the method of support used for the test objects in one case.

Although the potential benefits of sonic IR for practical NDE testing has been known to the NDE community for over a decade, many uncertainties with sonic IR still exist, such as the minimum sonic power and the effect of backing material. Attempts to explore the relationship between sonic energy and the amount of crack growth within a test sample were made by Chen *et al.* [44,51]. They subjected the samples to the sonic IR inspection technique under various conditions to see if there existed some conditions that could lead to further damage propagation in the form of crack extension [44]. Experimental results proved that sonic IR could cause cracks to extend under particular testing conditions, but the mechanism for crack growth under sonic IR conditions remained unknown. Various materials for use as backing materials and other inspection parameters were also explored in this paper. Results showed that the choice of backing material was important for sonic IR tests, and that high-density polyethylene (HDPE) had the potential to become the best choice to give consistent and repeatable results. Based on further research, they found that the extent of crack propagation strongly relied on the conditions under which the cracks were created [51]. Experimental results showed that cracks created under increasing stress intensity factors tended to grow less, and two hypotheses about the cause of this were discussed.

In order to find the minimum vibration demands for the detection of smaller cracks of more practical relevance, Morbidini and Cawley introduced a method to investigate the detection ability of fatigue cracks in metallic components using sonic IR [52]. The method relied on the validation of simple finite-element thermal models of the cracks. The experiment was accomplished on two beams: mild steel beams with two-dimensional cracks obtained in the low-cycle fatigue regime, as well as nickel-based super alloy beams with three-dimensional thumbnail cracks generated in the high-cycle fatigue regime. The strain required increased as the crack size decreased, and the desired temperature increased. For specimens with partially opened cracks, the predictions consistently overestimated the measured temperature profiles.

**Figure 19.**
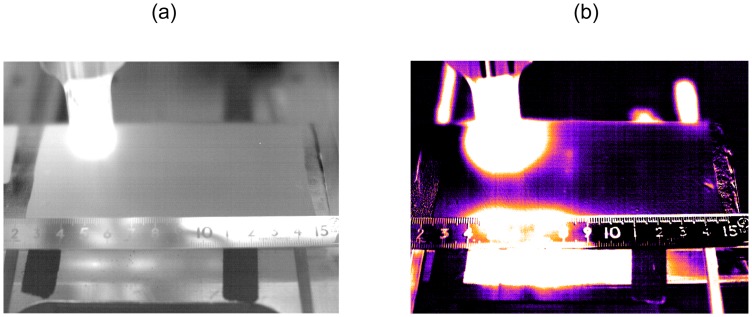
Results of sonic-IR testing for the stainless steel plate (reproduced from [49] with permission), (**a**) Raw infrared image; (**b**) self-reference lock-in image.

In the paper published by Xu *et al* [53], they examined a steel plate with fatigue crack and a juncture of carbon fiber composite that has been used in a space probe; a ceramic plate with a visible crack on the edge of the face was also tested, and the results were satisfying. The high speed, non-contact nature, the large imaging area and the sensitivity of the technique, especially the fact that it is suitable for cracks vertical to the structure's surface, made ultrasonic infrared thermal wave imaging an attractive NDE technique. It was significative for nondestructive testing in manufacturing and has application in aviation, cosmography and optoelectronics. For data processing, Sakagami *et al.* proposed the self-reference lock-in processing technique in 2009 [49]. They developed a sonic-IR system applied for the detection of artificially introduced stress corrosion cracking (SCC) flaws with a compact hand-held ultrasonic excitation unit and a micro-bolometer infrared camera. The self-reference lock-in data processing technique was based on the developed system and was employed to improve the signal/noise ratio of the infrared signals; the experimental results showed that noise reduction was useful for detecting small temperature increases at SCC flaws. Figure 19 shows the results obtained for the stainless steel plate with three SCC flaws, and it is found that heat generation is observed at the center SCC flaw by the improvement of the S/Nratio.

In the project *Investigation of Hybrid Acoustic-Infrared NDE Imaging Mechanisms* in 2010 [54], Han and Islam presented their research findings and studied several essential issues related to sonic infrared imaging: the relative motions between crack faces, the non-linear vibration behavior induced in the target materials and structures, via both experimental study and simulation. The aluminum samples fabricated with fatigue cracks were used for studying the heating mechanism related to different vibration modes in the samples. The zoom-in IR image with two spots and their corresponding temperature-time plots in a 300 ms-long ultrasound pulse showed that the temperature at the crack tip (red spot) was always higher than that at the open end of the crack (blue spot) during the excitation period before the temperatures dropped to the equilibrium value. The result verified that a highly non-linear situation existed indeed in the engaged system and that the frequency components and the number varied with time for open-closing or out-of-plane motion. It was illustrated that the amplitude of out-of-plane motion was typically bigger than that in open-closing motion.

**Figure 20.**
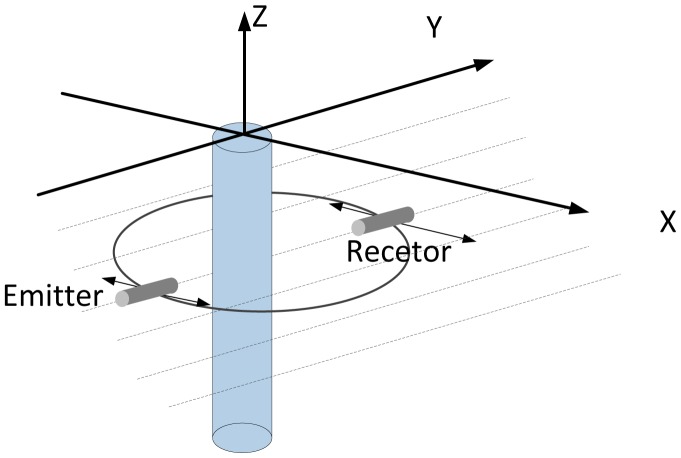
Arrangement of the transducers for the data capture for tomographic inversion.

Besides those above-mentioned efforts, in 2012, Gonzalez *et al.* developed an experimental system for data acquisition and algorithms for acoustic signal processing [48]. Pulse-echo and transmission methods, pulse echo lag techniques and a Cartesian scanner were used in this study. They managed to obtain images for analysis of the contrast of temperature in convective flows of air. Figure 20 sketches the set-up of the emitter and the receiver on a column of flow convective for the data acquisition. In summary, the developed system and technique complements traditional thermography and is helpful for its interpretation.

**Figure 21.**
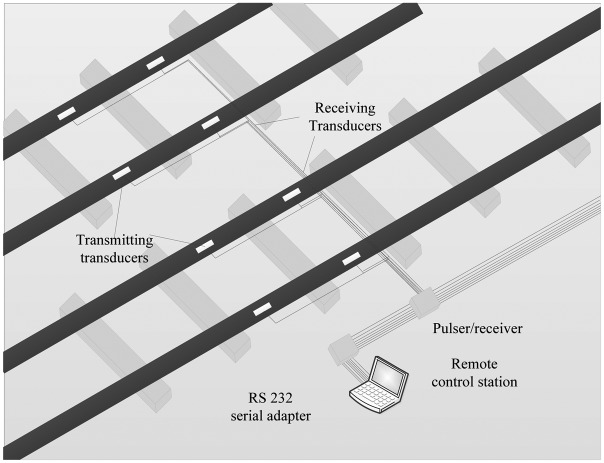
Arrangement of one of the 14 control sections.

It is known to all that the integrity and stability of civil infrastructure have far-reaching economic and social importance. After reviewing several theoretical and experimental works, Vangi and Virga made an effort in establishing a method to monitor internal stresses in continuous welded rails (CWR) and pointed out the main hindrances in employing ultrasonic techniques to the monitoring of rail [55]. The method presented here was based on the use of sub-surface longitudinal ultrasonic waves and was used to monitor thermally-induced loads on CWR; it was applied for a 3 km double rail track successfully. The application is illustrated in Figure 21.

As one of the pioneering groups in sonic IR technique, researchers at Wayne State University have also paid much attention to the damage assessment in civil structures [45,56]. Effective monitoring techniques for these large sized structures are necessary and urgent. To explore the effectiveness of a sonic IR technique on these structures, such as channels and beams, which were widely used in civil engineering structures, He and Han carried out experiments with steel C channel samples in 2009 [45].

Theoretical computing was also employed to assist the experiment. They made a further investigation of the heating patterns and some common fatigue cracks in other similar structures, which was published in 2012 [56]. The experiment on the fatigue crack around a rivet/bolt hole and welding joints proved the potential of this technology as a future tool for SHM.

### 3.3. Photothermal Methods

**Figure 22.**
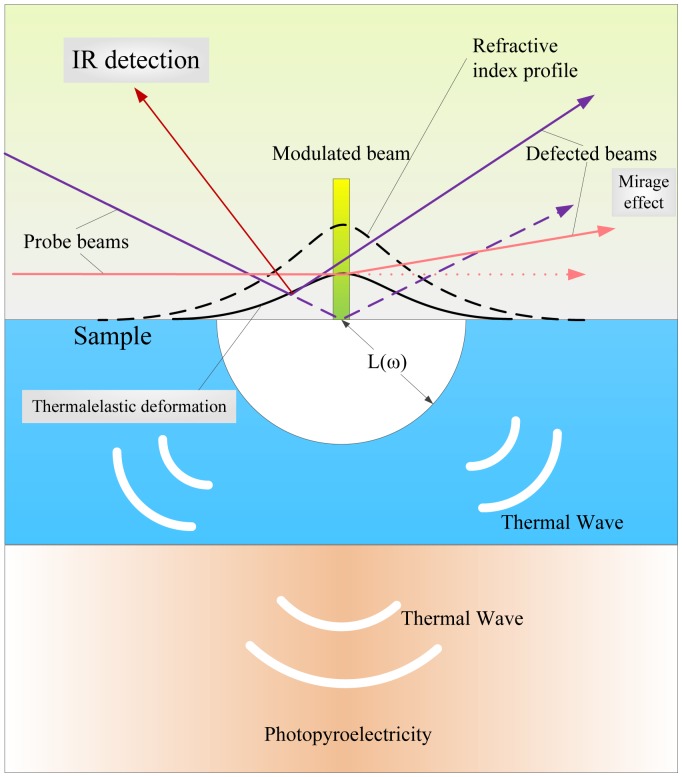
The photothermal effects in a sample illuminated by the pump beam.

The photothermal (PT) technique is based on the photothermal effect, initially a branch of the photoacoustic (PA) effect developed around 1880 [57]. The origin of optical-to-thermal energy conversion (photothermal or non-radiative) processes touches upon a great number of physical or chemical mechanisms, and photothermal techniques have become tools of increasing importance for the study of these types of energy conversion phenomena [58]. The launch of incident radiation in a sample causes heating due to the conversion of the absorbed light into thermal energy and, thus, results in various photothermal effects that make changes in both the material and the medium around it. Based on these ways of producing PT fields, as shown in Figure 22, photothermal radiometry (PTR), photothermal beam deflection, or PDS, and the photothermal displacement method were developed. As for the PA effect, which plays an important role in the development of photothermal science, as mentioned, will be discussed in more detail in next section.

**Figure 23.**
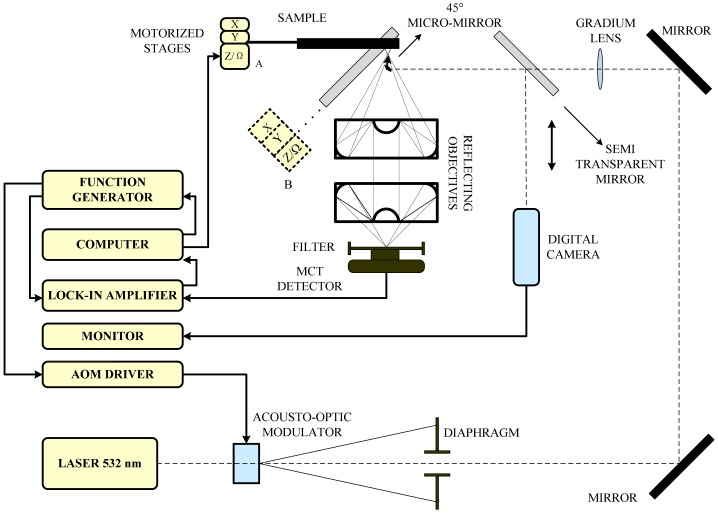
Schematic of the experimental system for photothermal radiometry of subsurface hairline manufacturing cracks.

PTR relies on the interaction of an intensity modulated laser-generated thermal wave with the crack, which results in changes of the amplitude and phase of the PTR signal. As for automotive components, such as sprockets, clutch plates and other parts usually under high strain, PTR also found application in the micro-crack diagnosis for green-state (unsintered) manufactured automotive parts [59]. In 2010, researchers used a modulated and focused laser beam to generate heat and a camera and a lock-in amplifier to record the changes of the detected signal, which is depicted in Figure 23. Statistical analysis of the experimental PTR frequency scanning phase data performed at sixteen points on the surface of five green sprockets has confirmed the excellent sensitivity (91%) of the method in detecting the presence of hairline (∼5–10 *μm)* cracks. It was assumed that the method was a good NDT technique for crack detection of green (unsintered) automotive parts and was very promising for feedback control in the metal forming process.

The photothermal beam deflection (PTBD) technique, also termed PDS, is based on the mirage effect, which means that the absorption of light causes a periodic heating and, hence, the thermal gradient in the medium causes bending of a light beam due to the changed refractive index. The PhD thesis work of Warrier dwelled on theoretical studies, as well as actual experiments with semiconductors and polymer materials using the PTBD technique [60], in which the photothermal deflection unit was automated and a theoretical model was developed for measuring the thermal diffusivity, minority carrier lifetime and surface recombination velocity of semiconductor thin films. The measurements were done with an objective of optimizing deposition conditions for obtaining device quality thin films. Besides, theoretical aspects of the application of photothermal techniques for solar cell analysis were elucidated.

Laser-spot thermography is a thermal NDE method for the detection of surface breaking cracks, primarily in metal components. It seems not to be unified on how laser-spot thermography is performed; however, the basis is always the use of a laser as a heat-generating source and measurement of the surface temperature a distance away from the laser-spot to reveal the cracks [61]. When there is a crack, the increased thermal impedance due to the restricted conduction of heat will result in alterations of surface temperature. A wealth of information could be found in the literature regarding laser-spot imaging, for example, using a flying spot scanner to detect cracks [62,63], in which the laser-spot was scanned across the sample with a point-reading from an infrared detector a distance way behind the heated spot. Additionally, Hermosilla-Lara *et al.* implemented two methods of thermal effect enhancement in order to improve the crack detection: image normalization and principal component analysis (PCA) [64]. In 2010, Weekes *et al.* carried out an experiment on a series of samples with different crack sizes to compare the detectability of fatigue cracks by thermosonic inspection and laser-spot thermography [61]. The main novelty of their work was that they used a series of point inspections with the raster and full-frame imaging rather than the typical use of a flying spot scanner. From the results obtained in the study, it seems that the detectability of fatigue cracks by laser-spot thermography increases almost linearly with the crack openings increasing.

Almond *et al.* developed two independent systems, respectively, with continuous wave (CW) laser and a pulsed laser beam to investigate crack detection by laser spot imaging thermography in 2007 [65,66]. A numerical model has also been developed to quantify the sensitivity of this technique to establish the limits of its performance. Experimental results indicated that that presence of cracks with openings ∼1 *μm* in metallic components could be identified. What is more, they found that pulsed laser heating simultaneously generated wide-band ultrasonic signals in the sample, so ultrasonic measurements with EMATs could also be done simultaneously to help detect cracks [66]. The effectiveness of this technique was proven by experiments on stainless steel and titanium samples.

For image processing techniques, the same group published an article on a second-derivative image processing method to extract micron-cracks after raster scanning a focused laser spot in 2010 [67]. Usually, raw infrared images are processed by methods, such as baseline subtraction, three-dimensional matched filtering or spatial derivation. The method in this paper was based on a full 3D “ghost point” (the concept was introduced to help to balance the heat flux because of the crack) heat transfer finite difference model. Although other features apart from the cracks may also be imaged and it is sensitive to thermal noise, like other second-derivative methods, it showed good sensitivity and high reliability for detecting near-surface micron-cracks.

Another study done by Vandone *et al.* [68] described an image processing algorithm aimed at automatically flagging the presence of the defect by analyzing the thermal data with two stages: firstly, correlated testing images to a baseline and using the correlation coefficient and/or the eccentricity to ascertain the presence of a defect; secondly, using the first and the second spatial derivatives of the surface temperature to identify the defect signature. The effectiveness of the proposed algorithm was experimentally verified by experiments with CFRP (carbon-fiber-reinforced polymer) and GFRP (glass-fiber-reinforced polymer) composite plates with induced defects. Besides, Laplacian and Roberts filters were also applied to thermal images to help to enhance the contrast and to locate and determine the size of the defects.

### 3.4. Photoacoustic Imaging

Photoacoustic (PA) imaging, also termed optoacoustic (OA) imaging, is a hybrid imaging system that combines the advantages of the high spatial resolution of ultrasound imaging and the high contrast of optical methods [69]. It is based on the PA effect, which was first reported by Alexander Graham Bell in 1880 [57]. He observed that audible sound could be created by illuminating an intermittent beam of sunlight onto a rubber sheet. The term, photoacoustic imaging, is used to describe a number of related imaging modes that exploit this effect to image objects with heterogeneous optical absorption.

**Figure 24.**
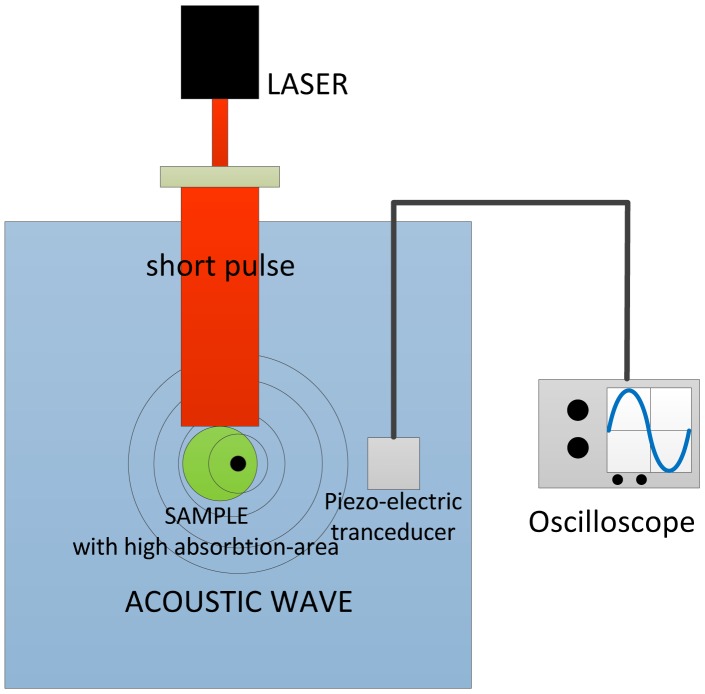
Schematic of a photoacoustic setup for bio-medical applications.

PA imaging was primarily developed for medical imaging and diagnosis, such as early cancer detection. It is found that cancer tissue absorbs more energy of the short electromagnetic pulses than the healthy tissue at specific wavelengths [70], leading to stronger ultrasound generation in malignant tissue. Early PA imaging utilized light pulses to irradiate a large area, and later on, laser was used to improve the performance. For biomedical applications, the sample is usually immersed in a tank filled with water (or another fluid) [69], as shown in Figure 24. Illumination from a short electromagnetic pulse (*i.e.*, a pulse laser) on the sample causes stress transients and then generates ultrasound waves, which propagate through water to the detector, such as a piezoelectric transducer.

Photoacoustic imaging has potential as a diagnostic method for prostate cancer in clinics, as well. Yaseen *et al.* developed a laser optoacoustic (OA) imaging system for the prostate (LOIS-P), which combined OA imaging with ultrasound [71]. The filtered radial back-projection (RBP) algorithm [72] was introduced to construct the two-dimensional tomographic OA images from the generated OA signals. The resolution was estimated to be 0.2 mm in the radial direction of the acoustic array. In the authors' point of view, the system was sensitive to the detection of early stage aggressively growing malignancies in the prostate. Additionally, they were devoted to further development toward a dual-modality OA/ultrasonic system for prostate diagnosis.

Actually, PA imaging can be divided into several categories: photoacoustic tomography (PAT), photoacoustic microscopy (PAM) and its variants [73]. These categories are, in some ways, all variations on a theme and more a consequence of the different imaging equipment that has emerged in the recent past than fundamental methodological differences. Because the primary purpose of this paper is to discuss the multi-wave and hybrid imaging methods for NDE and SHM applications, readers who are interested in PA imaging for biomedical diagnosis can refer to the heavily cited review articles [73,74].

The efforts carried out by Endoh *et al.* [75–77] broke the limitation that photoacoustic imaging with laser pulses is mainly applied to biological or medical samples and extended PAM in NDT for industrial applications. The PAM system presented in the literature [75] consisted of an argon ion laser, a computer-controlled closed-loop optical scanner and a PA cell. They experimented with pure aluminum plates [76] and made photoacoustic measurements with the modulation frequency changing. Two types of defects—tilted subsurface defects and wedge-type subsurface defects—were fabricated in the sample and amplitude images, as well as the phase images obtained by PAM and were processed simultaneously. Results indicated that the first type of defects were recognized in both kinds of images, and the wedged subsurface defects could be estimated from the amplitude PA images. In 2011, the same group performed shape measurement of the replicated weld defects by PAM [77]. The analytical result of the replica specimen by laser displacement measurement was compared with the PA amplitude images. As a result, the size of the weld defect estimated by both methods almost agreed in dimension.

**Figure 25.**
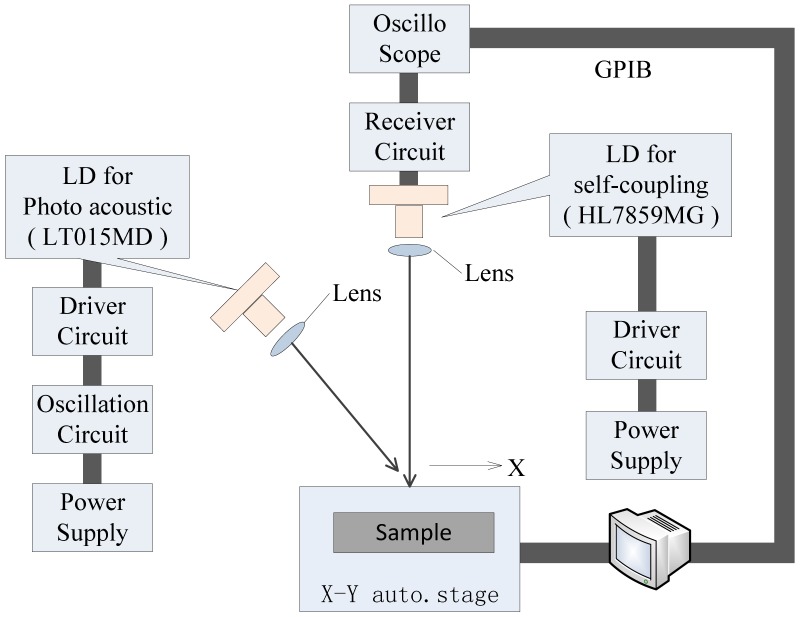
Schematic of the experimental setup using the photoacoustic and self-coupling effect to detect internal defects.

On the other hand, Oe *et al.*, in Japan, conducted research on internal defect detection using the photoacoustic and self-coupling effect [78]. The study of photoacoustic effect, self-coupling effect and edge effect offered fundamental interpretation in great detail. The developed detection system utilized a self-coupling sensor instead of an ultrasonic sensor with low sensitivity, which is shown in Figure 25.

Two LDs (low-power semiconductor laser) were used: a P-LD (photoacoustic LD) used for generation of the PA signal and an S-LD (self-coupling LD) used as a self-coupling vibration sensor. It is confirmed that the self-coupling sensor has no frequency limit, so researchers also studied the frequency dependence of the detection. From the results obtained in the investigation, they believed that the system had high spatial resolution and high sensitivity, e.g., it could detect small defects with dimensions down to 0.07 mm, and could detect smaller defects than the ultrasonic sensor.

**Figure 26.**
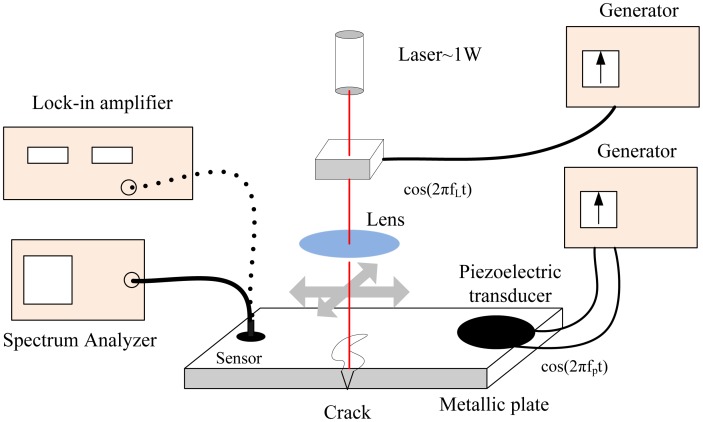
Experimental photoacoustic (PA)-acoustic imaging setup.

Zakrzewski *et al.* performed nonlinear imaging of cracks by a combining common PA imaging technique with additional acoustic loading [79]. Figure 26 shows the experimental setup: acoustic signals at two different fundamental frequencies are launched in the sample, one excited by the piezoelectric al transducer and the other by photoacoustic excitation with intensity-modulated laser radiation. Besides, several physical mechanisms responsible for the frequency-mixing processes in the vicinity of the crack were discussed. The contrast of the images at a mixed frequency is similar to that of the obtained linear photoacoustic (PA) images, indicating that optical, thermal and acoustical nonlinearities of the surface breaking cracks are not much higher than the nonlinearities of the intact material.

**Figure 27.**
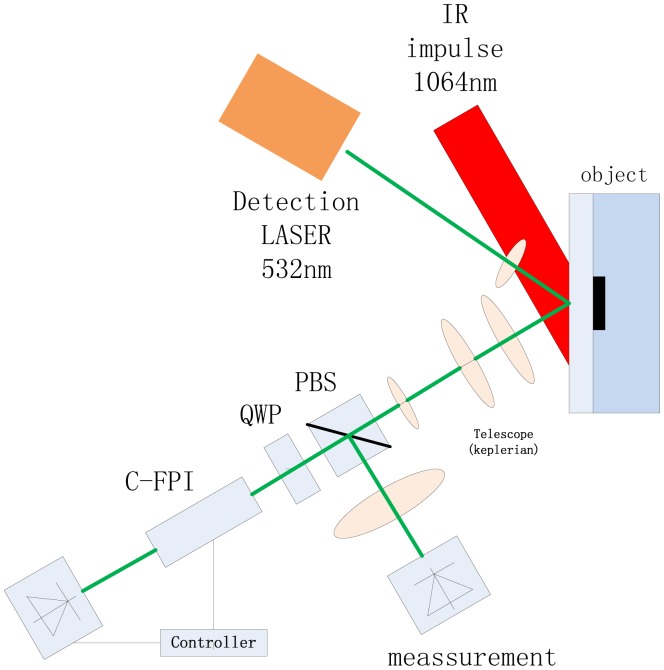
Schematic setup of the remote photoacoustic system.

**Figure 28.**
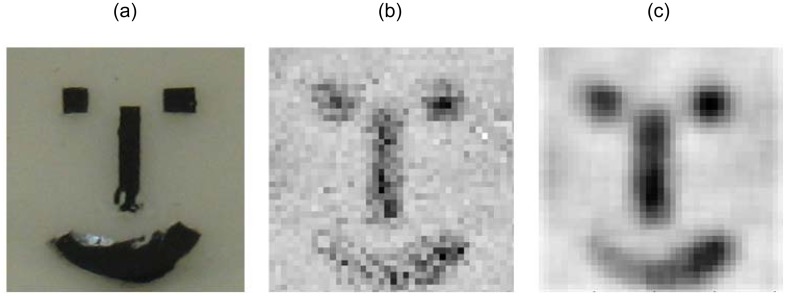
**(a)** Photograph of the measured object, back view; (**b**) wavelet filtered data at a time of 1.5 *μs;* (**c**) synthetic aperture focusing technique (SAFT) reconstruction of the data. The image quality is clearly enhanced (reproduced from [69] with permission).

For polymer materials, Hochreiner *et al.* proposed a remote contactless PA system based on a confocal Fabry-Prot Interferometer (CFPI) [80] to investigate the imaging of absorbing inclusions in semitransparent polymer samples by photoacoustic measurements [69]. The sample, consisting of a semitransparent polymer surface, black silicon glue and cast resin, is illuminated with a laser pulse, and the generated ultrasound waves are detected by a CFPI system, as illustrated in Figure 27. By scanning the sample, ultrasonic signals on the sample the surface could be obtained in two dimensions. Figure 28 displays the performance of the synthetic aperture focusing technique (SAFT) algorithm [81] as a promising method to compensate for blurring and to enhance the image quality. The group further studied the PA imaging reconstructed in three dimensions with similar equipments in 2011 [82]. After data acquisition, the absorbed energy density was reconstructed by utilizing a synthetic aperture focusing technique in the frequency domain (F-SAFT) algorithm. Their work proved the potential of PA imaging on material inspection in semitransparent solid materials.

**Figure 29.**
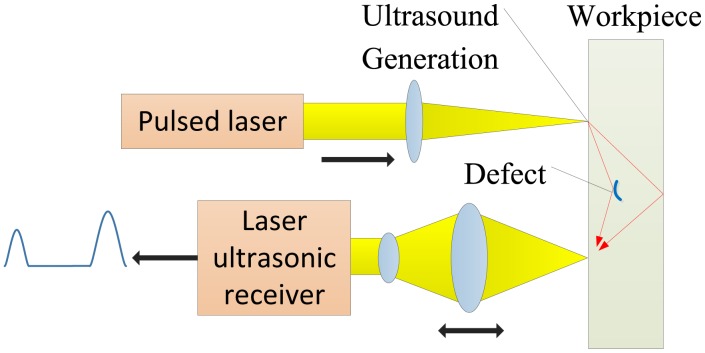
Schematic of laser ultrasonic detection of an internal defect.

There is another kind of detecting technique—laser ultrasonics (LUS) [83]—which shares a similar principle to PA imaging. LUS is a remote, non-contact technique for characterizing the mechanical properties of a material, relying on the effect that the irradiation of a short pulsed laser not only excites the ultrasonic source, but also generates compression, shear and surface waves on a range of materials. Figure 29 shows the schematic of the laser ultrasonic detection of internal defects. Research regarding the PA and LUS imaging was done by Burgholzer *et al.* with the simulated data and measurement data acquired with an interferometer setup [84]. In the authors' point of view, PA and LUS imaging modalities differ in where the conversion of optical into acoustic energy takes place. It is assumed that the source of the ultrasound wave in PA imaging is the investigated structure itself, while in LUS, the ultrasound pulses generated by the laser propagate into the sample, and the imaging is performed in pulse-echo mode, like in conventional ultrasound imaging. Both Fourier reconstruction and F-SAFT, which needs no interpolation, were mathematically applied in the study. As a result, the Fourier reconstruction method was mathematically equivalent to F-SAFT when the step size of the spatial discretization goes to zero.

**Figure 30.**
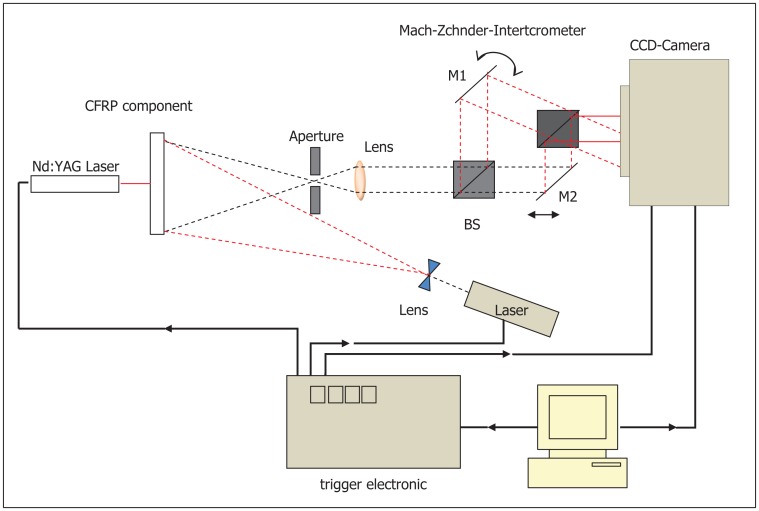
Laser-generated Lamb wave detection with a Mach-Zehnder receiver.

Nondestructive testing on composite structures is of great importance, since the most applied ultrasonic testing can hardly fulfill the demands. As a good extension of the conventional ultrasound technique, laser ultrasonic measurement presents many advantages, such as non-contact and enhanced resolution. In 2008, Kalms *et al.* expounded the principle of laser ultrasound in great detail and proposed a pulse-echo laser ultrasound system for the inspection of small complex CFRP and carbon fiber/polyphenylenesulphide (C/PPS) parts [85]. The performance of this system was demonstrated on parts of various shapes, thicknesses and compositions, and it proved laser ultrasound as a versatile method able to verify various defect sizes. Investigation of the laser-generated Lamb wave with a shearographic detection system is illustrated in Figure 30.

For the NDT of civil infrastructures, Abraham *et al.* [86] presented two possible applications of *in situ* LUS: one was the detection of voids in tendon ducts by impact echo diagnosis imagery in the frequency range of 1–60 kHz, and the other was the characterization of the cover of concrete structures using surface waves (SW) in the frequency range of 50–200 kHz. EMATs, mentioned previously, could also be introduced to detect the laser-generated ultrasound, and they showed favorable sensitivity when compared with laser-based interferometric detection [87], particularly where in-plane displacement was measured.

**Figure 31.**
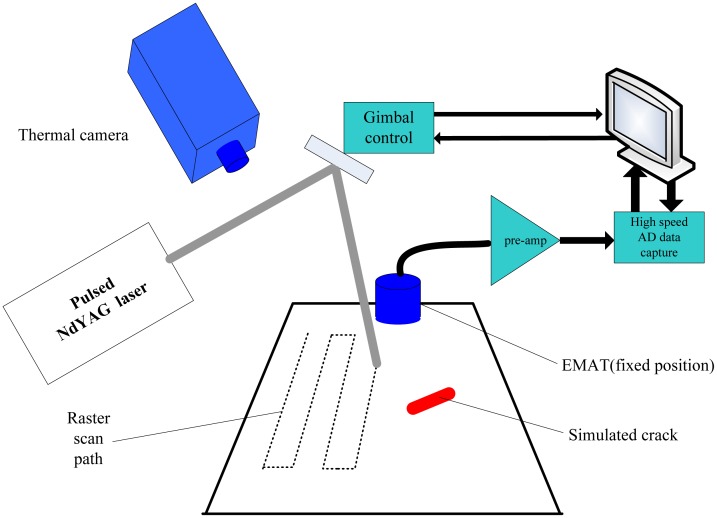
Schematic diagram of the experimental set-up used for non-contact ultrasonic and thermographic measurements.

In 2011, Palmer developed a combined ultrasound and thermography defect detection system using a raster scanned Q-switched laser as a source of heat that generates ultrasonic Lamb waves to identify surface breaking defects [88]. The experimental setup is shown below in the schematic diagram of Figure 31. In order to optimize source and detector positions around a defect, three-dimensional FEM of the interaction between Lamb waves and defects were studied and compared with the experimental data. It demonstrated that the realistic cracks, with gaping openings down to several microns, could be identified via the ultrasonic and thermography method.

### 3.5. Photoinductive Imaging

**Figure 32.**
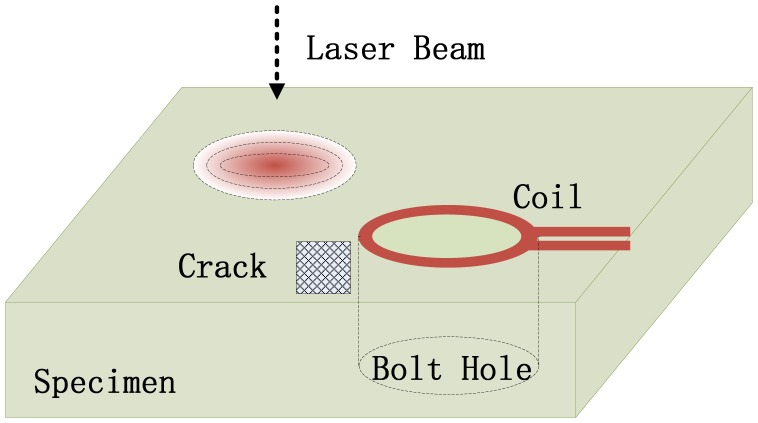
Inspection geometry of the photoinductive field measurement technique.

The photoinductive (PI) imaging method is a novel hybrid NDE technique that combines EC and laser-based thermal wave methods [89,90]. Figure 32 illustrates the physical principles of photoinductive imaging, which is similar to photofhermal imaging. A focused laser beam generates a localized hot spot on the specimen surface, and the temperature fluctuation causes variations of electrical conductivity, which, in turn, induces a change in the impedance of the eddy current probe in close proximity to the specimen surface. In a word, photoinductive (PI) imaging is a multiphysics sensing method. The effort made by Tai and Pan was mainly on the FEM simulation of the photoinductive (PI) imaging technique for bolt-hole crack inspection. They also discussed the effects of EC frequencies and the temperature of the thermal spot in the paper. It is shown that the photoinductive (PI) imaging technique is a novel sensing method for characterizing the geometric shape of cracks with high-resolution capability.

## 4. Hybrid Imaging

Multi-wave imaging refers to one form of wave energy serving as the excitation source of the other. Take the eddy current thermograph (ECT) as the example: the eddy current is used as the excitation to change the temperature in the specimen and, then, the infrared camera to image the temperature picture; the eddy current serves as the excitation source of the thermal energy. Furthermore, in the electromagnetic-acoustic (EM-A) techniques, the electromagnetic acoustic transducers generate ultrasonic waves via electromagnetic coupling between the transducer and the samples; the electromagnetic coupling is the excitation source of acoustic waves. However, hybrid imaging has a broader definition: that combing multiple imaging methods or two sources is a little different from multi-wave imaging. Like magneto-optic imaging, it combines the electromagnetic methods and the Faraday magneto-optic effect. Positron emission tomography/magnetic resonance (PET/MR) combines PET and MR.

### 4.1. Magneto-Optic Imaging

The magneto-optic imaging (MOI) technique invented by Shih and Fitzpatrick in the early 1990s can acquire fast imaging speed with high image resolution [91]. The main advantage of MOI is rapid inspection and ease of interpreting image data in contrast to complex impedance signals from conventional eddy current instruments [92]. The MOI device using eddy current induction techniques along with a magneto-optic sensor provides realistic, real-time images of both cracks and corrosion. The MOI was originally invented to inspect aluminum lap joints, and now, it is widely used in detecting surface and subsurface cracks and corrosion in aircraft skins [92]. Boeing and McDonnell Douglas published their procedures for the use of MOI in 1992. The military and other companies, such as Lockheed, followed in adopting MOI [93]. It is also used by the U.S. Air Force, NASA and many other organizations.

**Figure 33.**
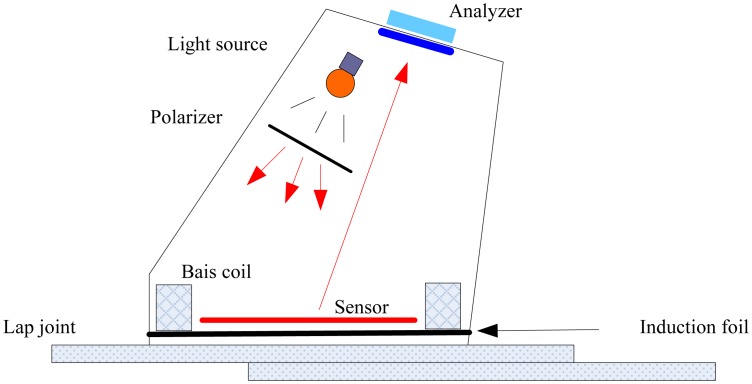
Basic schematic of magneto-optic imaging (MOI).

The basic schematic of MOI is shown in Figure 33. The MOI technique relies on exciting the aircraft skin by eddy current induction and measuring the normal magnetic field component using Faraday magneto-optic effect. The time varying magnetic field of the AC current passing through the planar induction foil induces a sheet of eddy current in the aircraft skin according to the law of electromagnetic induction, discovered also by Michael Faraday, like the magneto-optic effect. The presence of rivets or defects diverts the eddy current from its uniform flow and, hence, generates a magnetic field perpendicular to the surface of the aircraft skin. The normal magnetic field component is measured based on Faraday's magneto-optic effect, using a linearly polarized light transmitted in a magnetic garnet sensor. The light is transmitted parallel to the eddy current induced magnetic field, and according to the Faraday Effect, the light encounters rotation in the polarization plane depending on the magnetic field and the specific Faraday rotation value of the sensor material. As shown in Figure 33, the induction foil works as a reflector for incident light, and thus, the effective Faraday rotation angle is doubled, since the light passes through the sensor twice. Reflection-type magneto-optic imaging enhances the contrast of the resulting image. Perturbations in the magnetic field are monitored by measuring the rotation in light polarization. Images are obtained with pixel intensities dependent on the values of the normal magnetic field component. Magneto-optic imaging can be used to detect defects in both ferromagnetic and non-ferromagnetic materials [93].

For optimization of the MOI image, it should be modeled. The FE presents a powerful tool for exploring the interaction of the MOI source with material. M.M.ABD. Elnaby introduced a nodal finite element (FE) modeling of magneto-optic imaging for a complex geometry that is of interest to the aviation industry. Zeng and Deng develop a three-dimensional (3D) finite-element model for simulating the MOI performance; the model offers the capability to examine the effects of individual and multiple parameters on MOI performance [94]. P. Ramuhalli showed how to enhance the magneto-optic images in 2003 [95], Transient analysis using the nodal-based finite element method was used by I.M. Elshafiey to model the interaction between the MOI sensor and geometry under investigation. Transient FE analysis allows for the investigation of pulse width duration on inspection reliability. An animation of the magnetic field interaction with the material can be recorded to be thoroughly under the MOI phenomenon [93]. Deng and Liu presented an image processing and automated classification algorithm for MO image analysis and also provide a quantitative basis for characterizing these images [92].

In 2006, Fan and Deng *et al.* developed a real-time aircraft rivet imaging system based on the DSP TMS320C6000 platform and demonstrated in Detroit in 2007. This system cannot only reduce the detection variability, but also has the capability of segmentation, enhancement, quantization and classification. A magneto-optical microscope that uses the polarization modulation method has been developed in 2006 by Ishibashi and Takayuki for quantitative MO imaging [96]. In this technique, images of MO rotation and ellipticity are reconstructed from three images for different polarization states. The three polarization states are generated either by rotating a quarter-wave plate or by changing the voltage applied to a liquid crystal modulator (LCM). Real-time MO imaging with a rate of 1 frame/s is also achieved by using the LCM and a high-speed charge-coupled device camera. A new magneto-optical system developed by M. Baziljevich using a pump solenoid to rapidly excite the field coil is designed to expand the range of high speed real time magneto-optical imaging [97]. Together with careful modifications of the cryostat, to reduce eddy currents, ramping rates reaching 3,000 T/s have been achieved. Using a powerful laser as the light source, a custom designed optical assembly and a high speed digital camera, real time imaging rates up to 30,000 frames per second have been demonstrated.

**Figure 34.**
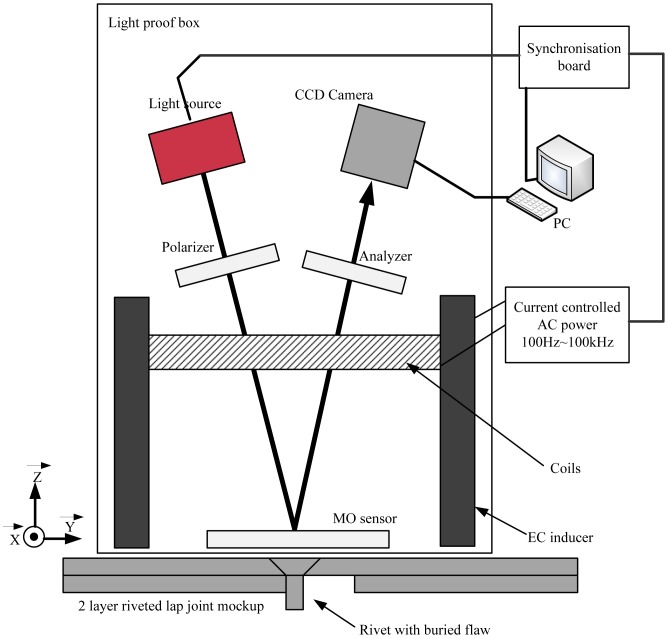
Schematic of the linear MO imager (LMOI).

One similar system, called LMOI (linear MO imager), was patented in Europe by Joubert *et al.*, which consist of the combination of a dedicated MO sensor featuring a linear and hysteresis-free magnetization loop, used with an original image acquisition system based on a stroboscopic approach and a specific high sensitivity eddy current inductor [98]. The schematic of the LMOI is shown in Figure 34, which is similar to the basic schematic of the MOI system.

In addition, Cheng *et al.* achieve an enhanced MOI system by using a laser to improve the sensitivity and image resolution in 2007 [99]. A high-sensitive scanning laser magneto-optical imaging system has also been developed by Murakami and Hironaru in 2010; the system is mainly composed of a laser source, galvanometers and a high-sensitive differential optical-detector [100]. Using the developed MO system, they have succeeded in the fast and quantitative imaging of a rotationally symmetric magnetic field distribution around a YBCOstrip line applied with DC-biased current and also succeeded in the detection of quantized fine signals corresponding to magnetic flux quantum generation in a superconducting loop of a YBCO Josephson vortex flow transistor.

#### 4.1.1. Magneto-Optical Kerr Effect Technique

The magneto-optical Kerr effect (MOKE) has important applications in modern information technology, especially in the design and manufacturing of high-density magneto-optical storage devices [101]. To increase the magnetic recording areal density, there is ongoing research into testing and characterization of different magnetic thin films [102]. One nondestructive approach to detect and characterize the in-depth defects in magnetic thin films is to study the saturation magnetization curves of the film [103]. The magnetic hardness coefficient obtained from the curves is related to defects in magnetic thin films and can be used to characterize the kinds of in-depth defects. A MOKE system can be used to measure the saturation magnetization curve, obtain the magnetic hardness coefficient and characterize the in-depth defects in magnetic films, fast and localized.

**Figure 35.**
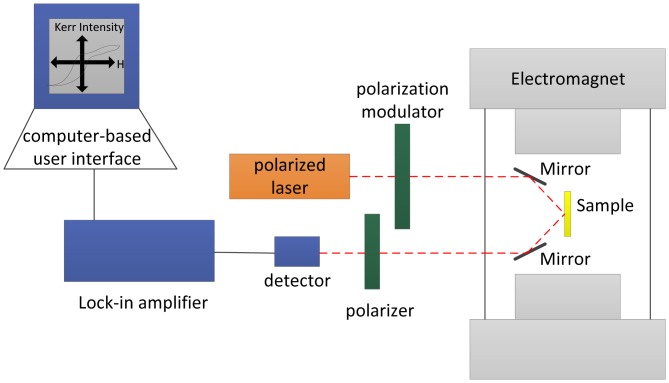
The configuration of the magneto-optical Kerr effect (MOKE) image measurement system.

The configuration of the MOKE image measurement system is shown in Figure 35.

The MOKE has become a standard technique for studying the magnetic properties of a variety of low dimensional systems like films, surfaces or multilayers. In this non-destructive surface sensitive technique, polarization-modulated laser light reflects from a magnetic surface (sample) in the presence of a sweeping magnetic field. Since light is an electromagnetic field, it is not surprising that the magnetic field of the sample interacts with the light to cause a very slight change in the light's polarization and ellipticity. We can measure these changes in the light as an intensity change through a nearly-crossed polarizer, recording the intensity as a function of the applied magnetic field. Three types of Kerr effect are known: polar, longitudinal and transversal; only polar and longitudinal Kerr effects are used in practice, because in transversal configuration, no depolarization takes place.

A method for imaging magnetic domains with a spatial resolution of less than 0.5 *μm* is described by Prakash kasiraj *et al.* [104]. The method employs the magneto-optical Kerr effect and is applicable for observing surface domain structures. Recent trends in the hard disk drive industry and severe competition in the development of new techniques and protocols for magnetic writing created strong demands for new efficient in-line inspection technologies. Kerr microscopy has always been considered as one of the most promising techniques for that purpose, due to the non-contact nature of the measurements. Vladimir V. Protopopov *et al.* have applied a heterodyne cross-polarized technique for imaging service magnetic tracks on magnetic disks by means of the longitudinal Kerr effect in 2006 [105]. The advantages of this technique over both the homodyne and direct detection techniques include higher sensitivity and lower noise in the output signal. A high-resolution magneto-optical imaging system is described by Daniel Golubchik *et al.* [106]. In this system, the magneto-optical Kerr effect is utilized for resolving individual flux quanta in a type II superconductor.

### 4.2. Others

Other multi-wave and hybrid methods in non-structural health monitoring share similar principles, such as positron emission tomography/computed tomography (PET/CT) and positron emission tomography/magnetic resonance (PET/MR) for human medicine and small animal imaging research [107]. PET radio pharmaceuticals enable the investigation of biochemical process at the cellular and molecular level *in vivo.* The goal of the PET scanner is to detect and count a large number of these annihilation photon pairs in coincidence to eventually reconstruct the image of the radioactivity distribution using tomographic techniques. In CT imaging, the transmission of X-rays from an external source through the subject is used to obtain an image of the tissue density. Tomographic, data are acquired by rotating the X-ray source around the subject, while recording the X-ray flux transmitted through the tissues in opposite detectors. Replacing CT by MR is considered to be the next evolutionary step in the field of hybrid imaging systems [108]. Unlike CT, MR does not measure the photon attenuation and, thus, does not provide easy access to this valuable information.

### 4.3. Multi-Modal Image Fusion

NDE systems are currently characterized by commercial systems that mostly use a single modality and depend on the subjective judgment of the human operator. Next-generation NDE systems are required to achieve high levels of automation and accuracy, and they are the focus of modern NDE research [109]. However, no single set of inspection parameters can provide robust information for most industrial applications. In this way, they require the use of more than one imaging/sensing modality to obtain enough information about the object or process under testing. At the same time, with the recent development in the field of sensor technology, there has been growing interest in the use of multiple sensors to increase the capabilities of intelligent machines and systems in a number of fields, such as surveillance, remote sensing, medical imaging, machine vision and military [110]. All of these have raised a need for processing techniques that efficiently integrate the information from multiple sensors into a single composition for further interpretation.

Image fusion is defined as the combination of a group of images with the objective of producing a single image of greater quality and reliability [111]. Additionally, the fusion process can be performed at different levels of information representation, namely, the pixel level, feature level and decision level [112]. As a necessary element in hybrid or array NDE systems, image fusion reduces the amount of data coming from multiple sensors and results in new images, which are more suitable for human/machine perception, and for further image-processing tasks, such as segmentation, object detection or target recognition. The prevalence of image fusion has also increased the demand for accurate methods of image-quality assessment in recent years, and a considerable amount of research has been done during the past decade.

The paper published by Eslami *et al.* presents a new family of perfect reconstruction, non-redundant and multiresolution geometric image transforms using the wavelet transform in conjunction with modified versions of DFB [113]. The proposed hybrid wavelet and DFB transform family provides visual and peak signal-to-noise ratio improvements over the wavelet and contourlet transforms.

Another study regarding an application directed at the eddy current (EC) inspection technique [109] was carried out by Algarni *et al.* It presents the application based on data fusion and the use of quality metrics. They implement the fusion algorithms by using the intensity hue saturation (IHS) transform, discrete wavelet transform (DWT) and IHS with shift invariant wavelet decomposition (SIDWT). The results of the objective evaluation are almost consistent with the subjective evaluation. Although each of the quality metrics gives a measure from a different viewpoint, all the metrics revealed the same trend in measuring the performance of the resulting fusion images. It is considered that advanced quality metrics can be used to cross the gap between subjective judgment depending on human operators and an automated system providing it based on objective measures.

In 2010, Shutao Li and Bin Yang proposed a hybrid multiresolution method by combining the stationary wavelet transform (SWT) with the nonsubsampled contourlet transform (NSCT) to perform image fusion [114]. Two methods, serial NSCT aiding SWT (SNAS) and serial SWT aiding NSCT (SSAN), are investigated and compared with some state-of-the-art methods, including NSCT, SWT, complex wavelet (CWT), curvelet (CVT) and wavelet-based contourlet (WBCT). The serial methods firstly decompose the source images into high-frequency coefficients and low-frequency coefficients using one transform. Then, the high-frequency coefficients are combined by selecting coefficients with the largest energy, and low-frequency coefficients are combined using the other transform-based image fusion methods. Experimental results have demonstrated that the SSAN method performs better than SNAS and the other individual multiresolution-based methods. However, the hybrid multiresolution method consumes more time than the SWT or the NSCT-based method, and this shortcoming should be resolved in the future with hardware implementation.

**Figure 36.**
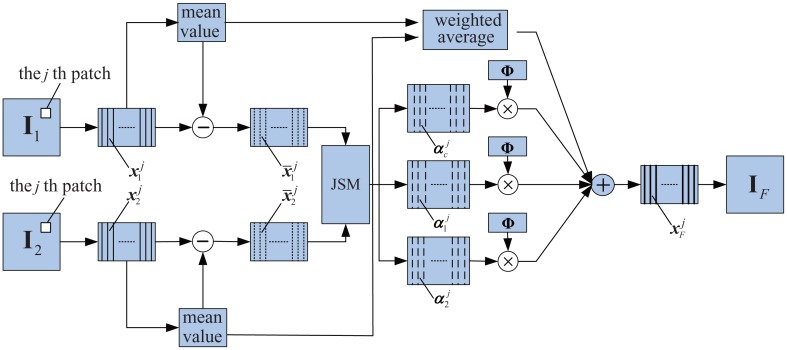
Diagram of the joint sparsity model (JSM)-based image fusion method.

It is considered that the difficulty of image fusion is how to separate the complementary information among the source images. Haitao Yin and Shutao Li then proposed a novel multimodal image fusion scheme based on the joint sparsity model (JSM) in 2011 [115]. The diagram of the proposed JSM-based image fusion method is depicted in Figure 36. Experiments on several different category source images engaging in different application fields, such as surveillance, weapon detection and medical diagnosis, are performed to demonstrate the performance of this method. The major contribution of the study was that they separated the complementary information of the multimodal images monitoring the same scene through the jointly sparse decomposition. Results demonstrate that multimodal images monitoring the same scene can be effectively separated through the jointly sparse decomposition, suggesting it is applicable to monitoring safety in cities and for making medical diagnoses.

As fusion of visible and infrared (IR) images and video sources is becoming increasingly important, many research studies have also been carried out on this topic. Cvejic and his collaborators [112,116–118] make their efforts to study the multimodal image fusion algorithm in the independent component analysis (ICA) [119] domain. They use segmentation to determine the most important regions in the input images and, consequently, fuse the ICA coefficients from given regions using the Piella fusion metric [120] to maximize the quality of the fused image. The proposed image fusion method was tested in the multimodal scenario with two input images: infrared and visible. Experimental results have shown that the method exhibits significantly higher performance, measured by the Piella and Petrovic fusion metric [121], than the basic ICA algorithm and is an improvement over other state-of-the-art algorithms.

**Figure 37.**
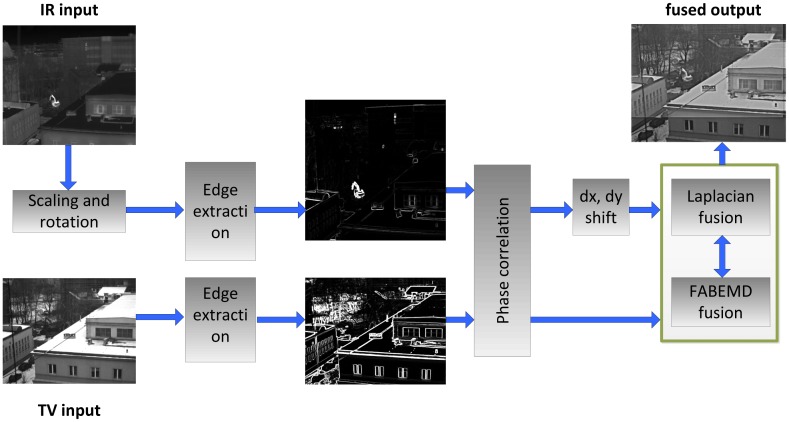
Video data processing flow implemented in the UFOsystem.

The region-based fusion methods, which can reduce the effect of noise, blurring effects and misregistration, are also explored. In 2009, T. Zaveri and M. Zaveri proposed a region-based image fusion method based on high boost filtering [122]. A nonparametric and region-based image fusion method was presented using the bootstrap sampling principle by M. Zribi in 2010 [123]. The article published by Tao *et al.* presented a dual-tree complex wavelet transform (DTCWT)-based fusion scheme with particle swarm optimization (PSO) [124] to automatically find the optimal contrast setting to obtain an optimal fused image. Experimental results demonstrate that the proposed fusion method performs better than the methods based on the DTCWT, the support value transform (SVT) and the nonsubsampled contourlet transform (NSCT), both visually and quantitatively. Additionally, Egfin Nirmala *et al.* proposed a novel method [125] for adaptive fusion of multimodal surveillance images, based on non-subsampled contourlet transform (NSCT), which has an improved performance over visual sensor networks. This method can compress the input data in the sampling process efficiently by using Compressive sensing (CS). It is interesting that Bartys *et al.* develop a real-time single FPGA -based (ALTERA Cyclone IV) multimodal image registering and fusion system called UFO [126]. This system is intended for low-cost and low-power applications for mobile and fixed platforms. The multimodal image processing chain implemented in the UFO system is shown in Figure 37, consisting of two phases: image registering and image fusion. The achieved experimental results have certified the excellent real-time performance of the system in challenging civil and military applications.

Recently, in order to better support more accurate clinical information for physicians to deal with medical diagnosis and evaluation, multimodality medical images have been needed, such as X-ray, computed tomography (CT), magnetic resonance imaging (MRI), positron emission tomography (PET) images, *etc.* [127]. The diversification of the typology of sensors for acquiring medical images provides abundant information that is useful for medical diagnosis. The information is complementary and occasionally conflicting. Therefore, the fusion of the multimodal medical images is necessary, and it has become a promising and very challenging research area in recent years [128].

**Figure 38.**
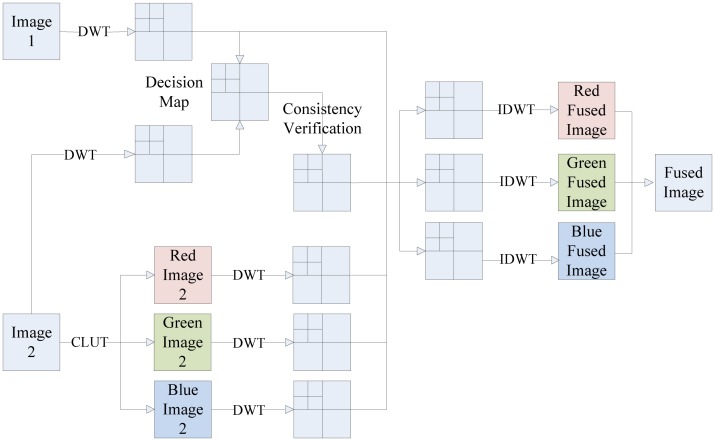
Fusion scheme of the novel Wavelet-based algorithm.

It is well established in the literature that the multiresolution analysis (MRA) [129] is the approach that best suits image fusion, and wavelet decomposition is the method that best fits the MRA approach regarding images. The paper published by Alfano *et at.* [130] presented a novel Wavelet-based algorithm to blend medical images according to the MRA approach, whose fusion scheme is shown in Figure 38. The algorithm aims to put the right “semantic” content in the fused image by applying two different quality indexes: variance and modulus maxima. Experimental results show that the proposed approach is encouraging in terms of both quantitative and qualitative evaluations.

Richa Singh at West Virginia University proposed a fusion algorithm that combines pairs of multispectral magnetic resonance imaging in 2009 [131]. This algorithm utilizes different features of the redundant discrete wavelet transform, mutual information based non-linear registration and entropy information to improve performance. This method has been evaluated on the BrainWeb database, and it has been proven that the proposed algorithm conserves important edge and spectral information without much spatial distortion. Yang in 2010 introduced a novel discrete wavelet transform (DWT)-based technique for medical image fusion [132]. After the source images are decomposed by the DWT, the coefficients of the low frequency portion and high frequency portions are performed with different fusion schemes. Finally, the fused image is constructed by the inverse DWT (IDWT) with all the combined coefficients. Experimental results on both simulated and real medical images show it to be an effective method and to have significant improvement over several conventional fusion methods.

## 5. Summary and Conclusions

In this article, we have summarized, thoroughly discussed and proposed the different concepts between multi-wave and hybrid imaging methods, which may provide a new direction for nondestructive evaluation and structural health monitoring. Besides, a comprehensive and up-to-date review of the latest research achievements in these techniques applied to various facets of NDE and SHM has also been conducted. Various modeling efforts, image and data processing techniques and other improvements to enhance image quality, e.g., resolution improvement, SNR enhancement and/or noise reduction, to achieve faster image acquisition are discussed for each category. The authors believe that multi-wave and hybrid imaging are now a fertile field from which new ideas and technologies are emerging. Those techniques have a bright future in the field of NDE and SHM applications and should be emphasized in future research and development in this community.

## Figures and Tables

**Figure 1.**
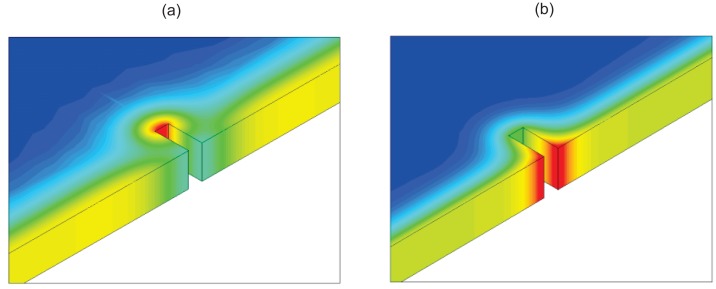
Calculated temperature distribution around a surface crack with a depth of 1 mm after 0.01 s of inductive heating: (**a**) penetration depth of the eddy current is 1 mm; (**b**) penetration depth of the eddy current is 0.1 mm [15].

## References

[1] Turqueti M., Kunin V., Cardoso B., Saniie J., Oruklu E. Acoustic Sensor Array for Sonic Imaging in Air.

[2] Tai C.C., Moulder J. (2000). Bolt-hole corner crack inspection using the photoinductive imaging method. J. Nondestruct. Eval..

[3] Fink M., Tanter M. (2010). Multiwave imaging and super resolution. Phys. Today.

[4] Wang X., Pang Y., Ku G., Xie X., Stoica G., Wang L.V. (2003). Non-invasive laser-induced photoacoustic tomography for structural and functional imaging of the brain *in vivo*. Nat. Biotechnol..

[5] Bercoff J., Tanter M., Fink M. (2004). Supersonic shear imaging: A new technique for soft tissue elasticity mapping. IEEE Trans. Ultrason. Ferroelectr. Freq. Control.

[6] Shinohara S., Sabra K., Genisson J.L., Fink M., Tanter M. (2010). Real-time visualization of muscle stiffness distribution with ultrasound SWI during muscle contractions. Muscle Nerve.

[7] Siakavellas N. A Proposal for Magneto-Thermal NDT in Conducting Materials.

[8] Zenzinger G., Bamberg J., Satzger W., Carl V. (2007). Thermographic crack detection by eddy current excitation. Nondestruct. Test. Eval..

[9] Tsopelas N., Siakavellas N. (2010). Eddy current thermography in circular aluminium plates for the experimental verification of an electromagnetic-thermal method for NDT. Nondestruct. Test. Eval..

[10] Tsopelas N., Siakavellas N. (2011). Experimental evaluation of electromagnetic-thermal non-destructive inspection by eddy current thermography in square aluminum plates. NDT&E Int..

[11] Abidin I.Z., Umar M.Z., Yusof M., Ibrahim M., Hamzah A., Salleh M. Advantages and Applications of Eddy Current Thermography Testing for Comprehensive and Reliable Defect Assessment.

[12] Kumar C.N.K., Krishnamurthy C.V., Maxfield B.W., Balasubramaniam K. (2008). Tone burst eddy-current thermography (tbet). AIP Conf. Proc..

[13] Krishnamurthy C., Balasubramaniam K., Biju N., Arafat M., Ganesan N. Tone Burst Eddy Current Thermography (TBET) for NDE Applications.

[14] Biju N., Ganesan N., Krishnamurthy C., Balasubramaniam K. (2012). Defect sizing simulation studies for the tone-burst eddy current thermography using genetic algorithm based inversion. J. Nondestruct. Eval..

[15] Oswald-Tranta B., Wally G. Thermo-Inductive Surface Crack Detection in Metallic Materials.

[16] Noethen M., Wolter K.J., Meyendorf N. Surface Crack Detection in Ferritic and Austenitic Steel Components Using Inductive Heated Thermography.

[17] Ramdane B., Trichet D., Belkadi M., Fouladgar J. (2010). 3-D numerical modelling of the thermo-inductive technique using shell elements. IEEE Trans. Magn..

[18] He Y., Tian G., Cheng L., Zhang H., Jackson P. Parameters Influence in Steel Corrosion Evaluation Using PEC Thermography.

[19] Zhang H., Tian G., He Y., Zuo X. Defect Depth Effects in Pulsed Eddy Current Thermography.

[20] Cheng L., Tian G. Pulsed Electromagnetic NDE for Defect Detection and Characterisation in Composites.

[21] Tsopelas N., Siakavellas N. (2006). Electromagnetic-thermal NDT in thin conducting plates. NDT&E Int..

[22] Tsopelas N., Siakavellas N. (2007). Performance of circular and square coils in electromagnetic-thermal non-destructive inspection. NDT&E Int..

[23] Tsopelas N., Siakavellas N. (2009). Improvements in electromagnetic-thermal non-destructive inspection by data processing. NDT&E Int..

[24] Chen Y.S., Hung Y.Y., Liu L. Electro-Thermography Technique for Nondestructive Testing (NDT) Applications.

[25] Weekes B., Almond D.P., Cawley P., Barden T. (2012). Eddy-current induced thermography-probability of detection study of small fatigue cracks in steel, titanium and nickel-based superalloy. NDT&E Int..

[26] Ren Z. (1998). Degenerated Whitney prism elements-general nodal and edge shell elements for field computation in thin structures. IEEE Trans. Magn..

[27] Cheng L., Tian G. (2011). Surface crack detection for carbon fiber reinforced plastic (CFRP) materials using pulsed eddy current thermography. IEEE Sens. J..

[28] Cheng L., Tian G. (2012). Comparison of nondestructive testing methods on detection of delaminations in composites. J. Sens..

[29] Yang Z., Ye P. Design of Electromagnetic Acoustic Transducer for Wheel Flaw Detection.

[30] Ogi H., Hirao M. (2003). EMATs for Science and Industry: Noncontacting Ultrasonic Measurements.

[31] Suchkov G. (2000). The main advantage of electromagnetic-acoustic testing technique. Russ. J. Nondestruct. Test..

[32] Wang S., Kang L., Xin P., Zhai G. Characteristic Research and Analysis of EMAT's Transduction Efficiency for Surface Detection of Aluminum Plate.

[33] Finkel P., Godinez V. (2004). Electromagnetic stimulation of the ultrasonic signal for nondestructive detection of ferromagnetic inclusions and flaws. IEEE Trans. Magn..

[34] Nagy P.B. (1998). Fatigue damage assessment by nonlinear ultrasonic materials characterization. Ultrasonics.

[35] MacLauchlan D.T., Clark S., Cox B., Doyle T., Grimmett B., Hancock J., Hour K., Rutherford C. Recent Advancements in the Application of EMATs to NDE.

[36] Ribichini R., Cegla F., Nagy P., Cawley P. (2012). Experimental and numerical evaluation of electromagnetic acoustic transducer performance on steel materials. NDT&E Int..

[37] Kang L., Mi W., Lv C., Wang S. Research on Weak Signal Detection Technique for Electromagnetic Ultrasonic Inspection System.

[38] Palmer S.B., Hernandez-Valle J.F., Dixon S. Electromagnetic Acoustic Transduction Using a Pulsed Electromagnet.

[39] Zhai G., Jiang T., Kang L., Wang S. A Method for Optimizing Excitation of Electromagnetic Ultrasonic Lamb Wave.

[40] Marklein R., Rahman M. Advanced Techniques for Modelling and Detection of Cracks in Hot Wire Steel.

[41] Vitaly I., Alexander V. Development of Instruments for NDT of Rail-Tracks with Use of Contactless Electromagnetic Acoustic Emission Transducers (EMATs).

[42] Zhu Y., Wang K., Kang L., Zhai G., Wang S. Rail Flaw Detection System Based on Electromagnetic Acoustic Technique.

[43] Miller W.O. An Evaluation of Sonic IR for NDE at Lawrence Livermore National.

[44] Kephart J., Chen J., Zhang H. Characterization of Crack Propagation During Sonic IR Inspection.

[45] He Q., Han X. Crack Detection Using Sonic Infrared Imaging in Steel Structures: Experiments and Theory of Heating Patterns.

[46] Favro L., Thomas R., Han X., Ouyang Z., Newaz G., Gentile D. (2001). Sonic infrared imaging of fatigue cracks. Int. J. Fatigue.

[47] Han X., Favro L.D., Ouyang Z., Thomas R.L. (2001). Thermosonics: Detecting cracks and adhesion defects using ultrasonic excitation and infrared imaging. J. Adhes..

[48] Gonzalez C., Reyna R., Chitty J. Ultrasonic Thermal Imaging.

[49] Sakagami T., Kuroki K., Kubo S., Katsumata R., Matsumoto Y., Harada Y. Detection of Stress Corrosion Cracking by Sonic-IR Technique.

[50] Favro L.D., Han X., Ouyang Z., Sun G., Sui H., Thomas R.L. IR Imaging of Cracks Excited by an Ultrasonic Pulse.

[51] Chen J.C., Kephart J., Lick K., Riddell W.T. (2007). Crack growth induced by sonic IR inspection. Nondestruct. Test. Eval..

[52] Morbidini M., Cawley P. (2009). The detectability of cracks using sonic IR. J. Appl. Phys..

[53] Xu W., Shen J., Zhang C., Tao N., Feng L. Ultrasonic Infrared Thermal Wave Nondestructive Evaluation for Crack Detection of Several Aerospace Materials.

[54] Han X., Islam M.S. Progress on Developing Acoustic-Infrared Imaging NDE Studying Motions in Crack Faces.

[55] Vangi D., Virga A. (2007). A practical application of ultrasonic thermal stress monitoring in continuous welded rails. Exp. Mech..

[56] He Q., Han X. (2012). Application of sonic IR imaging in civil structure health assurance. AIP Conf. Proc..

[57] Bell A.G. (1880). Upon the production and reproduction of sound by light. J. Soc. Telegr. Eng..

[58] Mandelis A. (2002). Photothermal diagnostic technologies. Opt. Photon. News..

[59] Tolev J., Mandelis A. (2010). Laser photothermal non-destructive inspection method for hairline crack detection in unsintered automotive parts: A statistical approach. NDT&E Int..

[60] Warrier A.R. (2010). Photothermal Beam Deflection for Non-Destructive Evaluation of Semiconductor Thin Films. Ph.D. Thesis.

[61] Weekes B., Cawley P., Almond D.P., Li T. (2010). The effect of crack opening on thermosonics and laser-spot thermography. AIP Conf. Proc..

[62] Krapez J., Gruss C., Huttner R., Lepoutre F., Legrandjacques L. (2001). La caméra photothermique. Partie I: Principe, modélisation, application à la détection de fissures. Instrumentation Mesure Métrologie (In French).

[63] Krapez J., Lepoutre F., Huttner R., Gruss C., Legrandjacques L., Piriou M., Gente D., Hermosilla-Lara S., Joubert P., Placko D. (2001). La caméra photothermique. Partie II: Applications industrielles, perspectives damélioration par un nouveau traitement dimage. Instrumentation Mesure Métrologie (In French).

[64] Hermosilla-Lara S., Joubert P.Y., Placko D., Lepoutre K., Piriou M. Enhancement of Open-Cracks Detection Using a Principal Component Analysis/Wavelet Technique in Photothermal Nondestructive Testing.

[65] Rashed A., Almond D.P., Rees D.A.S., Burrows S., Dixon S. (2007). Crack detection by laser spot imaging thermography. AIP Conf. Proc..

[66] Burrows S.E., Rashed A., Almond D.P., Dixon S. (2007). Combined laser spot imaging thermography and ultrasonic measurements for crack detection. Nondestruct. Test. Eval..

[67] Li T., Almond D.P., Rees D.A.S., Weekes B., Pickering S.G. (2010). Pulsed laser spot imaging thermography, modelling and experimental data. AIP Conf. Proc..

[68] Vandone A., Rizzo P., Vanali M. Image Processing for the Laser Spot Thermography of Composite Materials.

[69] Hochreiner A., Berer T., Burgholzer P. Remote Contactless Photoacoustic Imaging for Non Destructive Testing. http://www.ndt.net/article/ctc2010/papers/125.pdf.

[70] Chaudhary S.S., Mishra R.K., Swarup A., Thomas J.M. (1984). Dielectric properties of normal and malignant human breast tis-sue at radiowave and microwave frequencies. Indian J. Biochem. Biophys..

[71] Yaseen M.A., Fronheiser M., Bell B.A., Oraevsky A.A., Motamedi M., Ermilov S.A., Brecht H.P., Su R., Conjusteau A. (2010). Optoacoustic imaging of the prostate: Development toward image-guided biopsy. J. Biomed. Opt..

[72] Oraevsky A., Karabutov A., Vo-Dinh T. (2003). Optoacoustic Tomography. Biomedical Photonics Handbook.

[73] Beard P. (2011). Biomedical photoacoustic imaging. Interface Focus.

[74] Xu M., Wang L. (2006). Photoacoustic imaging in biomedicine. Rev. Sci. Instrum..

[75] Endoh H., Hiwatashi Y., Hoshimiya T. Nondestructive Evaluation of Simulated and Actual Surface Defects Using a Photoacoustic Microscope.

[76] Endoh H., Miyamoto K., Hiwatashi Y., Hoshimiya T. NDT of Tilted and Wedge-Type Subsurface Defects by Photoacoustic Microscopic Imaging.

[77] Kato R., Endoh H., Hoshimiya T. (2011). Nondestructive evaluation of weld defect by photoacoustic microscopy and its destructive inspection using replica. Jpn. J. Appl. Phys..

[78] Oe T., Nawa Y., Tsuda N., Yamada J. (2010). Nondestructive internal defect detection using photoacoustic and self-coupling effect. Electron. Commun. Jpn..

[79] Zakrzewski J., Chigarev N., Tournat V., Gusev V. (2010). Combined photoacoustic-acoustic technique for crack imaging. Int. J. Thermophys..

[80] Hercher M. (1968). The spherical mirror fabry-perot interferometer. Appl. Optics.

[81] Jensen J.A., Nikolov S.I., Gammelmark K.L., Pedersen M.H. (2006). Synthetic aperture ultrasound imaging. Ultrasonics.

[82] Berer T., Hochreiner A., Reitinger B., Grün H., Burgholzer P. (2011). Remote photoacoustic imaging for material inspection. J. Phys. Conf. Ser..

[83] Scruby C., Drain L. (1990). Laser Ultrasonics: Techniques and Applications.

[84] Burgholzer P., Berer T., Reitinger B., Nuster R., Paltauf G. Fourier Domain Reconstruction Methods in Laser Ultrasonics and Photoacoustic Imaging.

[85] Kalms M., Focke O., Kopylow C.V. Applications of Laser Ultrasound NDT Methods on Composite Structures in Aerospace Industry.

[86] Abraham O., Popovics J.S., Cottineau L.M., Durand O. (2011). Laser ultrasonics for civil engineering: Some applications in development for concrete non destructive testing. J. Phys. Conf. Ser..

[87] Murfin A.S., Soden R.A.J., Hatrick D., Dewhurst R.J. (2000). Laser-ultrasound detection systems: A comparative study with Rayleigh waves. Meas. Sci. Technol.

[88] Palmer S., Burrows S., Dixon S. Detection of Surface Breaking Cracks Using Thermographic and Non-Contact Ultrasonic Methods.

[89] Tai C.C., Pan Y.L. A Novel Multiphysics Sensoring Method Based on Thermal and EC Techniques and Its Application for Crack Inspection.

[90] Tai C.C., Pan Y.L. (2008). Finite element method simulation of photoinductive imaging for cracks. Prog. Electromagn. Res. Lett..

[91] Fitzpatrick G., Thome D., Skaugset R., Shih E., Shih W. (1993). Magneto-optic/eddy current imaging of aging aircraft: A new NDI technique. Mater. Eval..

[92] Deng Y., Liu X., Fan Y., Zeng Z., Udpa L., Shih W. (2006). Characterization of magneto-optic imaging data for aircraft inspection. IEEE Trans. Magn..

[93] Elshafiey I. Modeling of Magneto-Optic Eddy Current Inspection of Hidden Flaws in Aircraft Skin.

[94] Zeng Z., Liu X., Deng Y., Udpa L., Xuan L., Shih W., Fitzpatrick G. (2006). A parametric study of magneto-optic imaging using finite-element analysis applied to aircraft rivet site inspection. IEEE Trans. Magn..

[95] Ramuhalli P., Yuan F., Park U., Slade J., Udpa L. Enhancement of Magneto Optic images.

[96] Ishibashi T., Kuang Z. (2006). Magneto-optical imaging using polarization modulation mehod. Appl. Phys..

[97] Baziljevich M., Barness D., Sinvani M., Perel E., Shaulov A., Yeshurun Y. (2012). Magneto-optical system for high speed real time imaging. Rev. Sci. lustrum..

[98] Joubert P., Pinassaud J. (2006). Linear magneto-optic imager for non-destructive evaluation. Sens. Actuators A Phys..

[99] Cheng Y., Zhou Z., Tian G. (2007). Enhanced magneto-optic imaging system for nondestructive evaluation. NDT&E Int.

[100] Murakami H., Tonouchi M. (2010). High-sensitive scanning laser magneto-optical imaging system. Rev. Sci. Instrum..

[101] Zhu X.S., Zhao H.B., Zhou P., Xia G.Q., You H.Y., Zhang R.J., Li J., Wang S.Y., Ni W.M., Chen L.Y. (2003). A method to measure the two-dimensional image of magneto-optical Kerr effect. Rev. Sci. Instrum..

[102] Leong S.H., Wang J.P., Low T.S. (2000). Study of in-depth defects using magneto-optical Kerr effect by measuring the magnetic hardness coefficient in magnetic thin films. IEEE Trans. Magn..

[103] Wang J.P., Tan L.P., Liew T.Y.F., Low T.S., Wong H.L., Lee Y.K. Effects of DC Bias on the Thermal Stability of DC In-Line Sputtered CoCrTa/Cr Thin Film Media.

[104] Kasiraj P., Shelby R., Best J., Horne D.E. (1986). Magnetic domain imaging with a scanning Kerr effect microscope. IEEE Trans. Magn..

[105] Protopopov V.V., Lee S., Kwon Y., Cho S., Kim H., Chae J. (2006). Scanning heterodyne Kerr-effect microscope for imaging of magnetic tracks. Rev. Sci. Instrum.

[106] Golubchik D., Polturak E., Koren G., Lipson S.G. (2009). A high resolution magneto-optical system for imaging of individual magnetic flux quanta. Optics Expr..

[107] Fontaine R., Belanger F., Cadorette J., Leroux J.D., Martin J.P., Michaud J.B., Pratte J.F., Robert S., Lecomte R. (2005). Architecture of a dual-modality, high-resolution, fully digital positron emission tomography/computed tomography (PET/CT) scanner for small animal imaging. IEEE Trans Nucl. Sci..

[108] Salomon A., Goedicke A., Schweizer B., Aach T., Schulz V. (2011). Simultaneous reconstruction of activity and attenuation for PET/MR. IEEE Trans. Med. Imaging.

[109] Algarni A., Elshafiey I., Alkanhal M. Multimodal Image Fusion for Next Generation NDE Systems.

[110] Nirmala D., Paul B., Vaidehi V. A Novel Multimodal Image Fusion Method Using Shift Invariant Discrete Wavelet Transform and Support Vector Machines.

[111] Dixon T.D., Canga E.F., Noyes J.M., Troscianko T., Nikolov S.G., Bull D.R., Canagarajah C.N. (2006). Methods for the assessment of fused images. ACM Trans. Appl. Percept..

[112] Cvejic N., Bull D., Canagarajah C. (2007). Region-based multimodal image fusion using ICA bases. IEEE Sens. J..

[113] Eslami R., Radha H. New Image Transforms Using Hybrid Wavelets and Directional Filter Banks: Analysis and Design.

[114] Li S., Yang B. (2010). Hybrid multiresolution method for multisensor multimodal image fusion. IEEE Sens. J..

[115] Yin H., Li S. (2011). Multimodal image fusion with joint sparsity model. Opt. Eng..

[116] Cvejic N., Lewis J., Bull D., Canagarajah N. (2007). Adaptive region-based multimodal image fusion using ICA bases. IEEE Sens. J..

[117] Cvejic N., Bull D., Canagarajah N. Improving Performance of ICA Domain Multimodal Image Fusion in Presence of Noise.

[118] Cvejic N., Bull D., Canagarajah N. Multimodal Image Fusion in Sensor Networks using Independent Component Analysis.

[119] Hyvarinen A., Karhunen J., Oja E. (2001). Independent Component Analysis.

[120] Piella G. (2003). A general framework for multiresolution image fusion: From pixels to regions. Inf. Fusion.

[121] Petrović V., Xydeas C. (2005). Objective evaluation of signal-level image fusion performance. Opt. Eng..

[122] Zaveri T., Zaveri M. A Novel Region Based Image Fusion Method Using Highboost Filtering.

[123] Zribi M. (2010). Non-parametric and region-based image fusion with Bootstrap sampling. Inf. Fusion.

[124] Tao J., Li S., Yang B., Huang D.S., McGinnity M., Heutte L., Zhang X.P. (2010). Multimodal Image Fusion Algorithm Using Dual-Tree Complex Wavelet Transform and Particle Swarm Optimization. Advanced Intelligent Computing Theories and Applications.

[125] Nirmala D., Vignesh R., Vaidehi V. Multimodal Image Fusion in Visual Sensor Networks.

[126] Bartys M., Putz B., Antoniewicz A., Zbrzezny L. Real-Time Single FPGA-Based Multimodal Image Fusion System.

[127] Maes F., Vandermeulen D., Suetens P. (2003). Medical image registration using mutual information. Proc. IEEE..

[128] Zhu Y.M., Cochoff S.M. (2006). An object-oriented framework for medical image registration, fusion, and visualization. Comput. Methods Progr. Biomed..

[129] Piella G., Heijmans H. A New Quality Metric for Image Fusion.

[130] Alfano B., Ciampi M., Pietro G.D., Falcidieno B., Spagnuolo M., Avrithis Y., Kompatsiaris I., Buitelaar P. (2007). A Wavelet-Based Algorithm for Multimodal Medical Image Fusion. Semantic Multimedia.

[131] Singh R., Vatsa M., Noore A. Multimodal Medical Image Fusion Using Redundant Discrete Wavelet Transform.

[132] Yang Y. Multimodal Medical Image Fusion through a New DWT Based Technique.

